# Magnetic circular dichroism and computational study of mononuclear and dinuclear iron(iv) complexes†
†Electronic supplementary information (ESI) available: VT MCD spectra, VT and VTVH MCD intensity analysis of complex **1**, energies, *S*_*x*_, *S*_*z*_ values and Boltzmann populations of *S* = 1 magnetic sublevels as a function of the applied magnetic field, derivation of the excited states arising from the 1b_2_ → 2b_1_ transition, determination of the *C*-term sign of band 1 and the *E*(2e → 2a_1_) transitions for complex **1**, VTVH MCD spectra, VTVH simulations and the computed MCD spectrum of complex **2**. See DOI: 10.1039/c4sc03268c



**DOI:** 10.1039/c4sc03268c

**Published:** 2015-02-26

**Authors:** Shengfa Ye, Genqiang Xue, Itana Krivokapic, Taras Petrenko, Eckhard Bill, Lawrence Que Jr, Frank Neese

**Affiliations:** a Max-Planck Institut für Chemische Energiekonversion , Stiftstraße 34-36 , D-45470 Mülheim an der Ruhr , Germany . Email: shengfa.ye@cec.mpg.de ; Email: ebill@gwdg.de ; Email: frank.neese@cec.mpg.de; b Department of Chemistry , Center for Metals in Biocatalysis , University of Minnesota , 207 Pleasant St. SE , Minneapolis , Minnesota 55455 , USA . Email: larryque@umn.edu

## Abstract

High-valent iron(iv)-oxo species are key intermediates in the catalytic cycles of a range of O_2_-activating iron enzymes. This work presents a detailed study of the electronic structures of mononuclear ([Fe^IV^(O)(L)(NCMe)]^2+^, **1**, L = tris(3,5-dimethyl-4-methoxylpyridyl-2-methyl)amine) and dinuclear ([(L)Fe^IV^(O)(μ-O)Fe^IV^(OH)(L)]^3+^, **2**) iron(iv) complexes using absorption (ABS), magnetic circular dichroism (MCD) spectroscopy and wave-function-based quantum chemical calculations. For complex **1**, the experimental MCD spectra at 2–10 K are dominated by a broad positive band between 12 000 and 18 000 cm^–1^. As the temperature increases up to ∼20 K, this feature is gradually replaced by a derivative-shaped signal. The computed MCD spectra are in excellent agreement with experiment, which reproduce not only the excitation energies and the MCD signs of key transitions but also their temperature-dependent intensity variations. To further corroborate the assignments suggested by the calculations, the individual MCD sign for each transition is independently determined from the corresponding electron donating and accepting orbitals. Thus, unambiguous assignments can be made for the observed transitions in **1**. The ABS/MCD data of complex **2** exhibit ten features that are assigned as ligand-field transitions or oxo- or hydroxo-to-metal charge transfer bands, based on MCD/ABS intensity ratios, calculated excitation energies, polarizations, and MCD signs. In comparison with complex **1**, the electronic structure of the Fe^IV^ =O site is not significantly perturbed by the binding to another iron(iv) center. This may explain the experimental finding that complexes **1** and **2** have similar reactivities toward C–H bond activation and O-atom transfer.

## Abstract 2

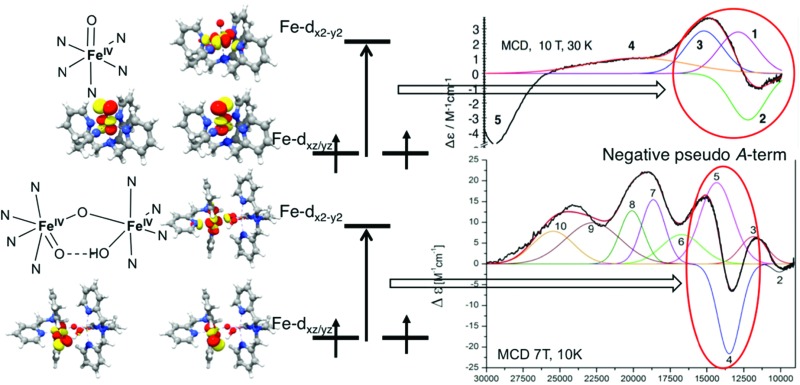
The electronic structures of mononuclear and dinuclear iron(iv) complexes are studied using magnetic circular dichroism and wavefunction-based *ab initio* methods, and then correlated with their similar reactivities toward H- and O-atom transfer.

## Introduction

In biology, a number of mono- and di-nuclear iron enzymes couple the activation of dioxygen to the selective functionalization of target C–H bonds.^1^ For mononuclear nonheme iron enzymes, C–H cleaving agents have been trapped and experimentally characterized as iron(iv)-oxo species in several α-ketoglutarate-dependent enzymes, namely taurine: α-ketoglutarate dioxygenase (TauD), prolyl-4-hydroxylase (P4H), and the halogenase CytC3.^2^ In the case of nonheme diiron enzymes, high-valent intermediates are also implicated in the catalytic cycles of soluble methane monooxygenase (sMMO), related bacterial multi-component monooxygenases, and fatty acid desaturases.^3^ In particular, the diiron(iv) intermediate **Q** of sMMO has been trapped and found to be responsible for the hydroxylation of methane;^4^ it was proposed to have an [Fe_2_^IV^(μ-O)_2_] diamond core on the basis of extended X-ray absorption fine structure (EXAFS) studies,^5^ and this structural hypothesis recently received strong support from a resonance Raman investigation of the intermediate.^6^

**Scheme 1:**
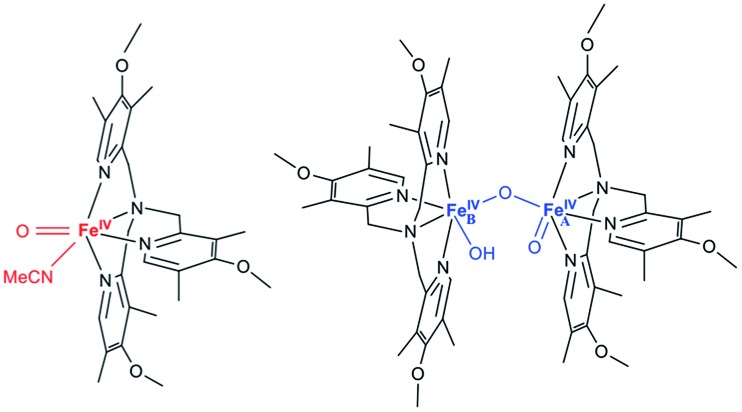
Proposed structures of complexes **1** (left) and **2** (right).

Over fifty mononuclear iron(iv)oxo model complexes have been prepared and characterized,^7^ for which complex **1** ^8^ serves as a representative example (Scheme 1), whereas synthetic analogs of high-valent diiron species are in contrast still quite rare.^9^ It was found in recent experiments that a complex with an [Fe_2_^IV^(μ-O)_2_] diamond core structure could be generated from open-core diiron(iv) complex **2** (Scheme 1) upon treatment with one equivalent of a strong acid.^9*b*^ Complexes **1** and **2** have been shown to cleave weak C–H bonds with similar efficiency. To understand their reactivity, the elucidation of their electronic structures is a prerequisite. Herein we present a detailed magnetic circular dichroism (MCD) study of complexes **1** and **2** in combination with wave-function-based *ab initio* calculations, and then correlate their electronic structures to the reactivities known about these two complexes. For clarity, hereafter we refer to the Fe^IV^ =O site of complex **2** as Fe_A_, and the Fe^IV^–OH site as Fe_B_.

Among electronic spectroscopy techniques, MCD spectroscopy has attracted tremendous interest because it is able to provide information about the geometric and electronic properties of the ground state such as oxidation and spin states, spin Hamiltonian parameters and coordination geometry as well as those of excited states. Thus, MCD serves as an invaluable link between ground state spectroscopy (electron paramagnetic resonance) and excited state spectroscopy (electronic absorption spectroscopy (ABS) and resonance Raman spectroscopy).

In MCD spectroscopy, the difference in the absorption (Δ*ε*) between the left (LCP) and right (RCP) circular polarized light of a sample is measured in the presence of a longitudinal magnetic field (relative to the light beam). MCD intensity arises from three different mechanisms designated as *A*-, *B*- and *C*-terms,^10^ and the following relationship is observed:^10*c*^

1

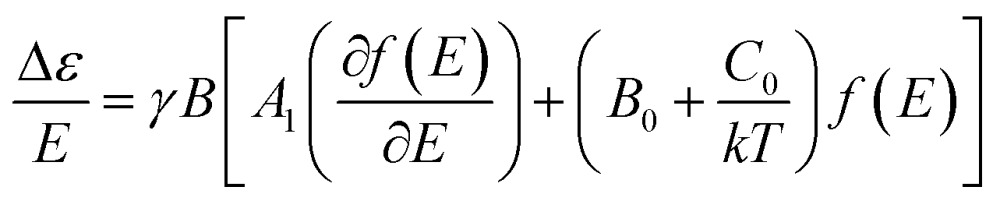




The *A*-term can only be non-zero if the excited state is degenerate and shows a derivative band shape, whereas the *B*-term originates from mixing of electronic states induced by the magnetic field. *C*-term contributions arise from the unequal Boltzmann populations of the ground state magnetic sublevels. Both *B*- and *C*-term signals have a usual absorption band shape. In contrast to *A*- and *B*-terms, the *C*-term intensity is temperature dependent and dominates the MCD spectrum at very low temperatures for paramagnetic molecules.

As the temperature decreases and/or the magnetic field increases, it is frequently observed that the MCD *C*-term signal is no longer linear with respect to *B*/*T* as implied by eqn (1) and levels off to its saturation limit. For *S* = 1/2 systems with orbitally non-degenerate ground states, the magnetization behavior follows a hyperbolic tangent and magnetization curves at all temperatures overlay. In the case of *S* > 1/2 systems, the magnetization curves do not superimpose and show a complicated nested behavior. The general theory to calculate the signs and intensities of MCD *C*-term transitions for *S* ≥ 1/2 has been simultaneously and independently worked out by Oganesyan *et al.* ^11^ and Neese and Solomon.^12^ Based on this formalism, one can determine ground-state spin-Hamiltonian (SH) parameters and the polarizations of the respective electronic transitions through fitting the nested magnetization curves obtained from variable-temperature variable-field (VTVH) MCD experiments. This method has been successfully applied to analyze MCD spectra of mono-^13^ as well as di-nuclear transition metal complexes.^14^

It has been shown that for paramagnetic systems VTVH MCD *C*-term intensity can be simulated using the following equation,^12^

2

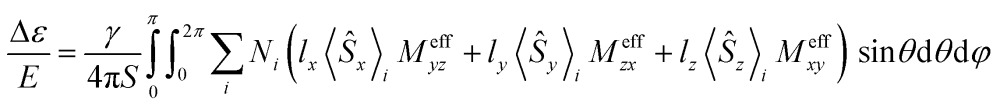

Here, 
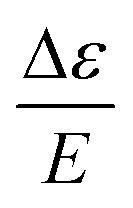
 is the MCD intensity, *γ* is a collection of constants, *S* is the total spin of the ground state, *N*_*i*_ is the Boltzmann population of the *i* ^th^ magnetic sublevel of the electronic ground state, *l*_*x*,*y*,*z*_ are the directional cosine values of the angles between the magnetic field and the molecular coordinate system, *ŝ*_*x*,*y*,*z*__*i*_ are the expectation values of the *x*,*y*,*z* component of the spin operator *ŝ* over the *i* ^th^ magnetic eigenstate, respectively. The *M*eff*vw*'s (*v*, *w* = *x*, *y*, *z*) are the effective transition dipole moment products and are given by^12^

3

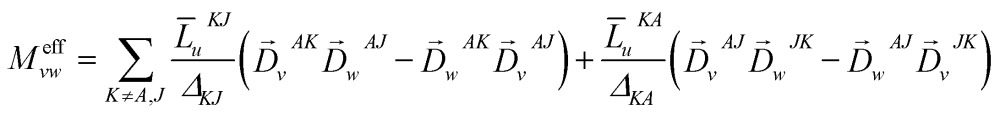

where *L*MN*t* is related to the reduced matrix element of spin–orbit coupling (SOC) operator, *Δ*_MN_ is the energy separation of state M relative to N, and *D*MN*t* is the electric transition dipole moment along *t*-direction. Moreover, the *x*, *y* and *z* fractional polarization factors can be computed as follows,
4

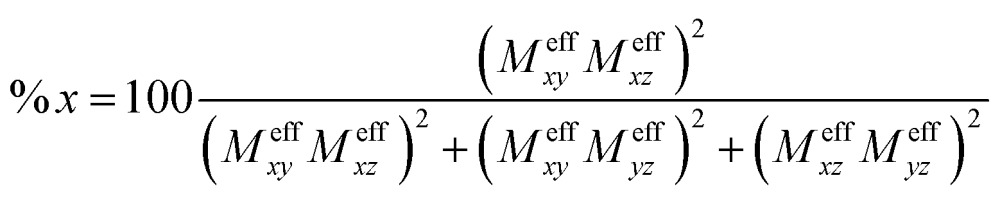

Cyclic permutation of the indices provides the appropriate expressions for the *y* and *z* polarized fractions of the intensity.

The SH parameters **g**, *D* and *E* enter the model through the spin expectation values and the Boltzmann populations of the various magnetic sublevels, and can therefore be calculated.

**Scheme 2:**
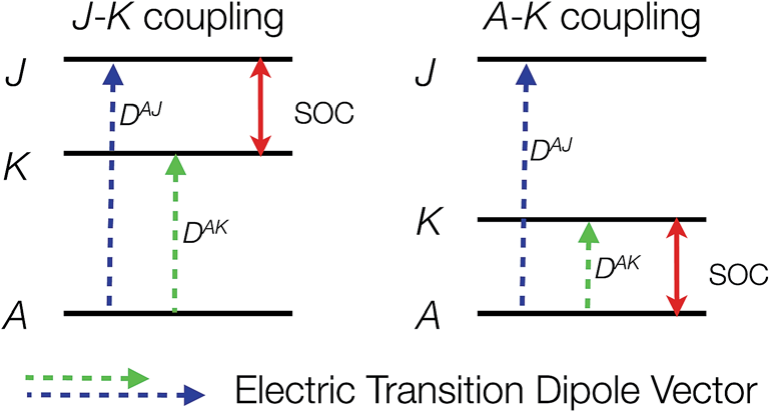
Mechanism for MCD intensity.

It is by no means straightforward to interpret MCD spectra and extract electronic structure information about excited states. Therefore, in most cases, MCD spectroscopy is employed as a complementary technique to conventional ABS spectroscopy for resolving and assigning electronic transitions.^15^ This lies in the fact that MCD spectra typically exhibit higher resolution than corresponding ABS spectra especially in the presence of multiple overlapping absorption bands, because MCD features are signed quantities. In order to fully exploit the information content present in the MCD spectrum, one can determine the individual MCD signs of electronic transitions of interest. This will definitely lower the possibility of erroneous band assignments and provide more insight into electronic structures. However, only a few examples concerning the determination of MCD signs have been reported so far,^16^ due to the stricter selection rules for MCD compared to ABS spectroscopy. It has been shown that for a system with orbitally non-degenerate ground and excited states, the MCD *C*-term intensity arises from SOC between the excited states *J* and *K* (*J*–*K* coupling) and between ground state *A* and excited state *K* (*A*–*K* coupling) (Scheme 2).^11a,12^ Nonzero *C*-term intensity obtained by the *J*–*K* coupling mechanism requires that the electronic transitions *A* → *J* and *A* → *K* be polarized in different directions perpendicular to the SOC vector that couples *J* with *K*. If both transitions can be observed in the MCD spectra, they usually show a derivative-shaped band (called a pseudo-*A* term), because the two MCD signals in principle have opposite signs and similar excitation energies. The absolute signs of the *A* → *J* and *A* → *K* transitions depend on the symmetry of the states *A*, *J*, *K*. If the transition is dominated by a single excitation, the MCD signs can be determined by the symmetry of the electron donating orbital (EDO) and the electron accepting orbital (EAO).

The best way to quantitatively interpret experimental MCD spectra is to compute them using quantum chemical approaches. The theoretical prediction of MCD spectra has been proven to be rather challenging. Based on the formulation of *A*-, *B*-, and *C*-terms by Stephens *et al.*, perturbational calculations of these terms were attempted.^8,9^ Recently, Seth, Ziegler and co-workers reported an elegant formulation of MCD *C*-term intensity based on linear response perturbation expressions of three MCD terms (eqn (1)) using time-dependent density functional theory.^17^ This work presents definite progress in the computational approach to MCD. However, it does not solve the fundamental problem that with DFT approaches one is unable to represent the all-important magnetic sublevels *M*_s_ = *S*, *S* – 1, …, –*S* of a system with a total spin *S* explicitly. Furthermore, for a high-spin system (*S* > 1/2) the very limited accuracy of DFT in the prediction of zero-field splitting (ZFS) parameters will affect MCD calculations. In addition, these approaches are only applicable in the linear response regime with respect to the magnetic field strength as implied by eqn (1). More importantly, single determinant methods such as density functional theory fail in the description of multiplet effects or double excitations, both of which are prevalent in the optical spectra of transition metal complexes. In this regard, one has to resort to multi-reference approaches^18^ such as the complete active space self-consistent field (CASSCF)^19^ and second-order *N*-electron valence perturbation theory (NEVPT2).^20^ Our approach to calculate MCD signs and intensities is to directly estimate the difference between LCP and RCP transition probabilities in the presence of a homogeneous external magnetic field using exact diagonalization rather than perturbation theory. Specifically, transition energies and intensities for LCP and RCP light are computed using ground and excited state wavefunctions obtained from quasi-degenerate perturbation theory (QDPT) treatment, which explicitly accounts for SOC, spin–spin coupling (SSC), and Zeeman interactions.^21^ The method was tested successfully for diatomic molecules as well as for simple transition metal complexes.^21^ Here we report the first application of this methodology to more complex mono- and dinuclear transition metal compounds.

## Experimental section

MCD samples of complexes **1** and **2** were prepared with 1 : 4 acetonitrile–butyronitrile (MeCN–PrCN). Upon freezing, this solvent mixture becomes a transparent glass, making it suitable for MCD measurement. Anhydrous MeCN were purchased from Aldrich and used as received. PrCN (99%+) purchased from Aldrich was purified and dried according to reported procedures.^22^ 2.2 mM [Fe^II^(L)(MeCN)_2_](OTf)_2_ in 1 : 4 MeCN–PrCN was maintained at –60 °C and treated with 2 equivalents of 2-(*tert*-butylsulfonyl)-iodosylbenzene, similar to a reported procedure for preparation of oxoiron(iv) complexes.^23^ Complex **1** formed in 2 minutes with ∼90% yield, as indicated by its absorption at 720 nm.^24^ 4.4 mM solution of **2** in MeCN was prepared according to a reported procedure,^9*c*^ then diluted with 4 equal volumes of cold PrCN. The solutions were transferred to pre-cooled MCD holders with pre-cooled syringes, and then frozen in liquid nitrogen.

MCD experiments were carried out with an Olis DSM17 CD spectrapolarimeter while the sample was placed in the Oxford cryostat Spectromag SM4000. The temperature range was from 2 K to 60 K and the energy range from 5000 cm^–1^ (2000 nm) to 30 000 cm^–1^ (333 nm). At the peak positions we carried out VTVH MCD experiments in which the MCD intensity is measured at the fixed wavelength as a function of temperature and magnetic field. The results are presented as plot of MCD intensity *vs. βB*/*kT*, where *β* is the Bohr magneton, *k* is Boltzmann's constant, *B* is strength of the applied magnetic field and *T* is absolute temperature.

All calculations were performed with the ORCA program package.^25^ Given the size of the complexes under investigation, the substituents on the pyridine ring were replaced by hydrogen atoms, *viz.* an unsubstituted TPA ligand (TPA = tris(2-pyridyl-methyl)amine) was employed in the calculations. Geometry optimizations were carried out using the hybrid B3LYP density functional^26^ along with the semi-empirical van der Waals corrections due to Grimme.^27^ Def2-TZVP(–f)^28^ basis sets on Fe, O, and N, and def2-SV(P)^29^ on the remaining atoms were used. The density fitting and “chain of spheres” (RIJCOSX)^30^ approximations were employed to accelerate the calculations in conjunction with the auxiliary basis sets def2-TZV/J.^31^ Solvation effects were taken into account by using the conductor like screening model (COSMO).^32^ Consistent with experiment, acetonitrile was selected as the solvent in the calculations.

The CASSCF/NEVPT2 approach was used to compute the MCD spectra of complexes **1**. In the CASSCF calculations, an active space consisting of twelve electrons in the five Fe-3d based molecular orbitals (MOs), the three oxo-p orbitals and the bonding counterpart of the Fe-d_*x* ^2^–*y* ^2^_ orbital (CAS(12,9)) was chosen. However, the CASSCF results erroneously predict a quintet ground state, because CASSCF is designed to mainly capture static correlation energies and hence lacks balanced treatments between static and dynamic correlation effects. Using NEVPT2, dynamic correlation effects were explicitly introduced. It turns out that the NEVPT2 calculations deliver the correct spin-state energetics for complex **1**; therefore, in the following we will only discuss the NEVPT2 results.

If a similar active space (CAS(12,9)) for each Fe^IV^ site of complex **2** were employed, the active space for the entire complex (CAS(24,18)) would be too large to render usual CASSCF calculations feasible. Using density matrix renormalization group (DMRG)^33^ approach, exploring CASSCF (CAS(24,18)) calculations on a model complex in which TPA is replaced with four NH_3_ ligands suffered from severe convergence problems. More importantly, current DMRG calculations only deliver excitation energies and are not able to provide the information about the nature of excited states and hence the assignment of transitions. Given these issues, complete active space configuration interaction (CASCI) is the only method of choice, since time-dependent DFT (TD-DFT) yields qualitatively unreasonable results (*vide infra*). To circumvent the problem of the size of active spaces, we carried out CASCI calculations with a series of active spaces. CAS(12,12) is composed of the five d-orbitals in each iron center and the two p-orbitals of the bridging oxo ligand (O_B_) that form π-bonds with the *t*_2g_-derived d-orbitals of Fe_B_, and CAS(14,13) consists of the five d-orbitals in each iron center and the three p-orbitals of the terminal oxo group (O_T_).

## Results and discussion

### Complex **1**

#### ABS and MCD spectroscopy

**Fig. 1:**
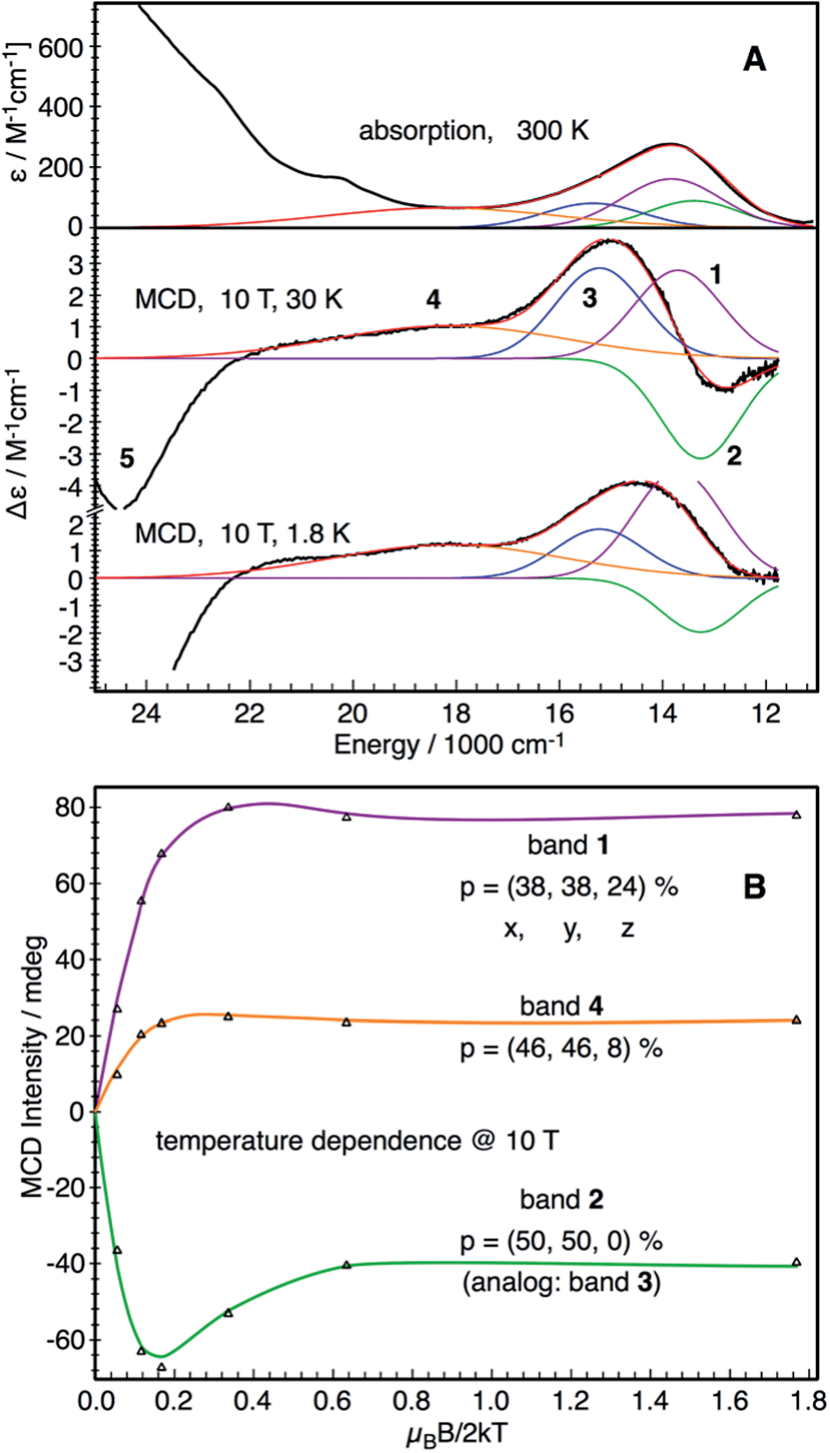
Experimental electronic absorption and MCD spectra of complex **1** along with the simultaneous Gaussian deconvolution (A), and VT MCD magnetization curves for bands 1–4 (B). The experimental points in (B) are obtained from the VT MCD spectra recorded at 10 T field (Fig. S1†), and the lines are derived from a SH simulation^12^ with the fixed SH parameters *D* = 28 cm^–1^, *E*/*D* = 0. The effective transition dipole moment products *M*eff*vw* obtained from the global parameter optimization yield fractional polarization factors as indicated in (B).

The electronic absorption spectrum of complex **1** exhibits a broad feature centered around 13 800 cm^–1^ in the near-IR region (Fig. 1A). However, the MCD intensities of the corresponding transitions display a rather complicated temperature dependence. In the temperature range 2–10 K a broad positive band dominates the MCD spectra, which gradually changes to a derivative-shaped signal as the temperature increases up to ∼20 K (Fig. S1†). The simultaneous Gaussian deconvolution of the absorption and MCD spectra reveal that this feature can be successfully modeled by three transitions, a positive band (band 1) and a pseudo *A*-term signal (bands 2 and 3). At higher temperatures, the intensities of the three bands decrease due to the usual MCD *C*-term behavior with increasing temperatures (Fig. S1†). The absorption and MCD features of complex **1** and the temperature dependent behavior of their MCD intensities are similar to those observed for [Fe^IV^(O)(TMC)(NCMe)]^2+^, [Fe^IV^(O)(TMC)(OC(O)CF_3_)]^2+^ and [Fe^IV^(O)(N4Py)]^2+^ (TMC = 1,4,8,11-tetramethyl-1,4,8,11-tetraazacyclotetradecane, N4Py = *N*-(bis(2-pyridyl)-methyl)-*N*,*N*-bis-(2-pyridylmethyl)-amine).^34^ In addition, a broad band centered around 18 200 cm^–1^ (band 4) and another intense negative band at 24 200 cm^–1^ (band 5) are found for complex **1**. Our variable temperature (VT) (Fig. 1B and S2†) and VTVH MCD intensity analysis (Fig. S3†) show that bands 2, 3, 4 and 5 are essentially *x*,*y*-polarized, whereas band 1 has no well-defined polarization property.

#### Electronic structure

**Fig. 2:**
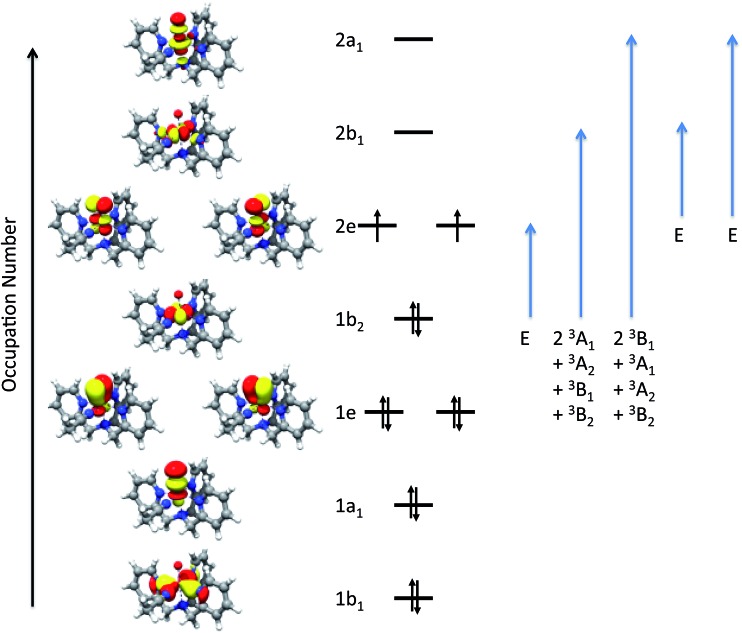
CASSCF active orbitals of complex **1** and the spin-allowed d–d excitations. The indicated orbital occupation pattern corresponds to the ^3^ *A*_2_ ground state under the effective symmetry of *C*_4*v*_.

As analyzed in an earlier study, the iron center in complex **1** is situated in a distorted octahedral environment with a characteristic Fe–oxo bond distance of 1.67 Å.^8^ Similar to other well-characterized ferryl species,^7^ this short metal–ligand bond distance reflects the rather covalent nature of the Fe–oxo interaction, which consists of two half π-bonds (involving the Fe-d_*xz*,*yz*_ and O-p_*x*,*y*_ orbitals) and one σ-bond (involving the Fe-d_*z* ^2^_ and O-p_*z*_ orbital).^35^ The orbital occupation pattern of the triplet ground state for complex **1** can be described as σ(O-p_*z*_)^2^π(O-p_*x*,*y*_)^4^δ(Fe-d_*xy*_)^2^π*(Fe-d_*xz*,*yz*_)^2^σ*(Fe-d_*x* ^2^–*y* ^2^_)^0^σ*(Fe-d_*z* ^2^_)^0^ (Fig. 2). The key ligand field excited states are as follows: the excitation from the doubly occupied molecular orbital (DOMO) 1b_2_(Fe-d_*xy*_) to nearly degenerate singly occupied molecular orbitals (SOMOs) 2e(Fe-d_*xz*,*yz*_) leads to a ^3^ *E*(1b_2_ → 2e) excited state. Due to multiplet effects, the excitations from 1b_2_(Fe-d_*xy*_) to the virtual molecular orbitals (VMOs) 2b_1_(Fe-d_*x* ^2^–y^2^_) and 2a_1_(Fe-d_*z* ^2^_) result in a series of excited states as shown in Fig. 2.^36^ Specifically, if the two electrons in the 2e(Fe-d_*xz*,*yz*_)-set have parallel spin, the excited states are of ^3^ *A*_1_(1b_2_ → 2b_1_) (× 2), and ^3^ *B*_1_(1b_2_ → 2a_1_) (× 2) symmetry. On the other hand, if the two electrons in the 2e(Fe-d_*xz*,*yz*_)-set have opposite spin, the excited states of ^3^ *A*_2_(1b_2_ → 2b_1_), ^3^ *B*_1_(1b_2_ → 2b_1_), ^3^ *B*_2_(1b_2_ → 2b_1_), and ^3^ *A*_1_(1b_2_ → 2a_1_), ^3^ *A*_2_(1b_2_ → 2a_1_), ^3^ *B*_1_(1b_2_ → 2a_1_) symmetry, respectively, arise. Promotion of one electron from the 2e(Fe-d_*xz*,*yz*_)-set into the 2b_1_(Fe-d_*x* ^2^–y^2^_) and 2a_1_(Fe-d_*z* ^2^_) orbitals gives rise to ^3^ *E*(2e → 2b_1_) and ^3^ *E*(2e → 2a_1_) excited states.

#### Assignment

**Fig. 3:**
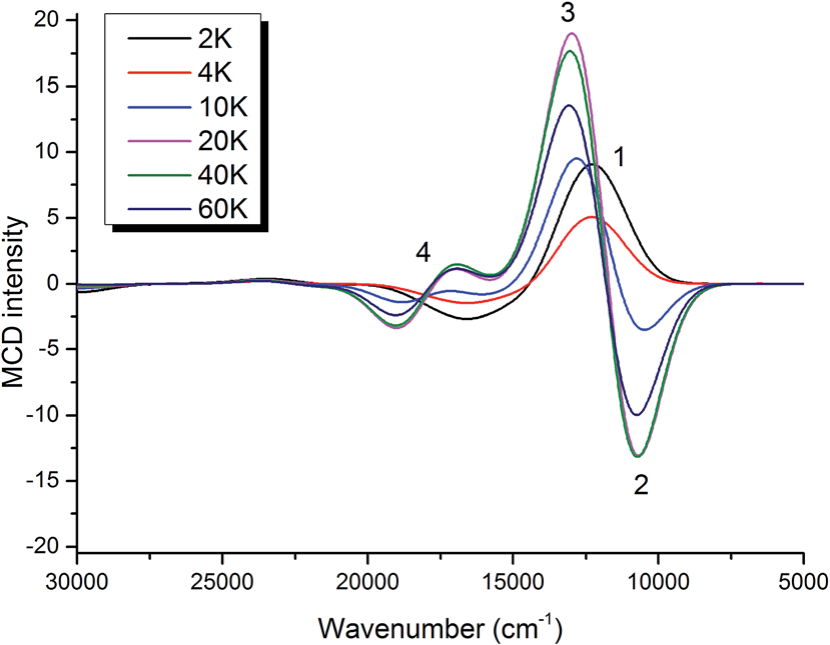
Computed MCD spectra of complex **1** at 10 T and indicated temperatures. To simulate Gaussian broadening the full-width-half-maximum of 2500 cm^–1^ is employed.

TD-DFT is a method based on the linear response formalism starting from the single Kohn–Sham determinant of the ground state, and is hence unable to properly describe multiplet effects and spin-coupling. As a consequence, it has been reported that the TD-DFT calculations on [Fe^IV^(O)(NH_3_)_4_(H_2_O)]^2+^ only deliver two roots for the excitations of (1b_2_ → 2b_1_) and (1b_2_ → 2a_1_), instead of five as required by spin-coupling algebra.^36^ To circumvent this problem, explicitly spin-coupled multi-reference approaches such as CASSCF and NEVPT2 have to be employed. Our NEVPT2 calculations not only yield quite accurate ligand field transition energies with a deviation of ∼3000 cm^–1^ relative to experiment (Table 1), but also successfully reproduce the MCD intensity changes observed experimentally at variable temperatures (Fig. 3), both lending credence to the proposed assignments of the transitions. It should be kept in mind that the computational error is within the standard error range of a good quantum chemical approach. The large deviation found for charge transfer (CT) transitions mainly originates from the fact that the limited active space is employed in the CASSCF calculations (*vide infra*).

**Table 1:** Comparison of experimental and calculated excitation energies of key transitions along with the assignments for **1** and **2**

Complex **1**				Complex **2**			
Band	Exp. (cm^–1^)	Calc. (cm^–1^)	Assignment	Band	Exp. (cm^–1^)	Calc. (cm^–1^)	Assignment
				1	7280	8270	Fe_B_ *t*_2g_ → d_*x* ^2^–*y* ^2^_
				2	9700	9320	Fe_B_ *t*_2g_ → d_*x* ^2^–*y* ^2^_
1	13 270	13 170	^3^ *A*_2_(1b_2_ → 2b_1_) d_*xy*_ → d_*x* ^2^–*y* ^2^_	3	11 600	10 630	Fe_A_ d_*xy*_ → d_*x* ^2^–*y* ^2^_
2, 3	13 700, 15 220	11 840, 12 060	^3^ *E*(2e → 2b_1_) d_*xz*,*yz*_ → d_*x* ^2^–*y* ^2^_	4	13 460	11 440	Fe_A_ d_*yz*_ → d_*x* ^2^–*y* ^2^_
		13 320, 13 400	^3^ *E*(1b_2_ → 2e) d_*xy*_ → d_*xz*,*yz*_	5	14 970	12 840	Fe_A_ d_*xz*_ → d_*x* ^2^–*y* ^2^_
				6	16 850	14 740	p_O_H__ → Fe_B_ LMCT
4	18 200	17 960, 18 050	^3^ *E*(2e → 2a_1_) d_*xz*,*yz*_ → d_*z* ^2^_	7	18 450	16 890	Fe_A_, Fe_B_ *t*_2g_ → d_*z* ^2^_
				8	19 880	18 540	Fe_A_, Fe_B_ *t*_2g_ → d_*z* ^2^_
5	24 200	∼30 000	LMCT	9	22 600	21 270	LMCT
				10	25 380	23 110	LMCT

As analyzed previously for related complexes by Solomon and co-workers,^34^ the temperature-dependent MCD *C*-term intensity of complex **1** stems from the distinct polarizations of the electronic transitions corresponding to bands 1, 2 and 3. The MCD intensity is proportional to *S*_*u*_ *M*eff*vw*, where *M*eff*vw* represents the effective transition dipole moment product. Complex **1** has been experimentally shown to have a positive axial ZFS parameter *D* of 28 cm^–1^ and the rhombicity parameter *E*/*D* close to zero.^8^ At 2 K, only the lowest energy magnetic sublevel (*M*_s_ = 0) is populated; thus, *S*_*x*_, *S*_*y*_ = 0, and *S*_*z*_ = 0 (Fig. S4 and S5†). Consequently, transitions polarized along the *z*-direction gain MCD *C*-term intensities. By contrast, around 20 K, the *M*_s_ = –1 magnetic sublevel becomes considerably populated, and for this magnetic sublevel *S*_*x*_, *S*_*y*_ = 0 and *S*_*z*_ = –1 (Fig. S4 and S5†). Therefore, *x*,*y*-polarized transitions acquire MCD intensities. At even higher temperatures the intensities for both types of transitions decrease because the MCD *C*-term intensity is inversely proportional to the temperature.

Based on the intensity variation with different temperatures, band 1 was assigned as the *z*-polarized ^3^ *A*_2_(1b_2_ → 2b_1_) transition under the effective *C*_4*v*_ symmetry.^34^ However, this is a two-electron excitation (Fig. S6†) and hence has vanishing intensity. Lowing the symmetry from *C*_4*v*_ to *C*_*s*_, the actual symmetry of complex **1**, allows band 1 to “borrow” intensity from intense transitions. The calculations suggest that it predominantly mixes with bands 2 and 3 due to the energetic proximity, in consistent with the polarization deduced from the VT and VTVH MCD analysis. For bands 2 and 3, we can unambiguously assign them as the two components of ^3^ *E*(2e → 2b_1_) based on their polarizations, the computed transition energies (Table 1) and the individual signs of the two transitions of ^3^ *E*(2e → 2b_1_) (*vide infra*). In the previous MCD study on related complexes,^34^ bands 2 and 3 were assigned as the ^3^ *E*(1b_2_ → 2e) and ^3^ *E*(2e → 2b_1_) transitions, respectively. However, our theoretical results reveal that the oscillator strength of the former transition is one to two orders of magnitude lower than that of the latter, and that the intensity of band 3, despite being opposite in sign, is nearly the same as that of band 2 over a large range of temperatures (>20 K) as shown in Fig. 3.

**Fig. 4:**
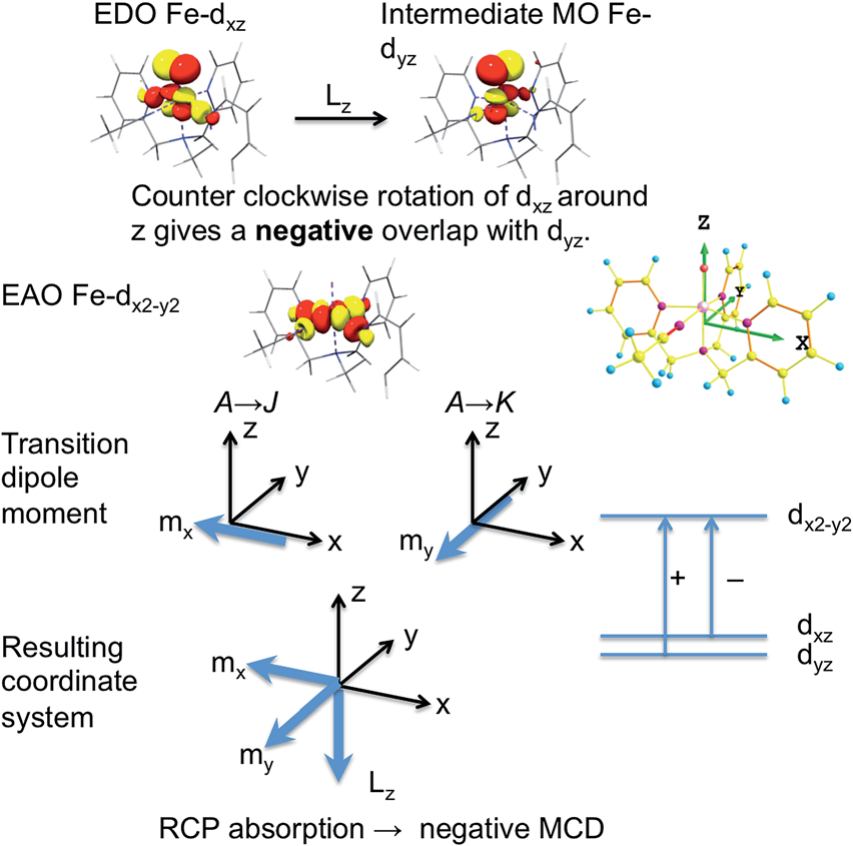
Graphical prediction of the *C*-term sign for the *E*_*x*_(d_*xz*_ → d_*x* ^2^–*y* ^2^_) transition.

To further corroborate our assignments, we determine the individual signs of the two transitions of ^3^ *E*(2e → 2b_1_). The pseudo *A*-term of bands 2 and 3 arises from SOC between the two constituent transitions of *E*_*x*_(d_*xz*_ → d_*x* ^2^–*y* ^2^_) and *E*_*y*_(d_*yz*_ → d_*x* ^2^–*y* ^2^_) (*J*–*K* coupling). Because of the difference in donor abilities between the pyridine ligand in TPA and MeCN, the former transition lies lower in energy. In the case of the lower energy transition, *J* = *E*_*x*_(d_*xz*_ → d_*x* ^2^–*y* ^2^_), and *K* = *E*_*y*_(d_*yz*_ → d_*x* ^2^–*y* ^2^_). Because pure d–d transitions are parity forbidden, the transition dipole moments of the d_*xz*,*yz*_ → d_*x* ^2^–*y* ^2^_ transitions, in fact, originate from the “overlap” of the ligand N-p orbitals as the iron center moves out of the equatorial plane slightly. The transition density defines the transition dipole moments of *E*_*x*_(d_*xz*_ → d_*x* ^2^–*y* ^2^_) and *E*_*y*_(d_*yz*_ → d_*x* ^2^–*y* ^2^_) pointing along –*x* and –*y* directions, respectively (Fig. 4). Counter-clockwise rotation of the d_*xz*_ orbital into the intermediate d_*yz*_ orbital around *z* leads to a negative overlap, and hence *L*_*z*_^*KJ*^ < 0. If one neglects the contribution from other excited states except the transition *A* → *K* = *E*_*y*_, the effective transition dipole moment product for *A* → *J* = *E*_*x*_ can be expressed as
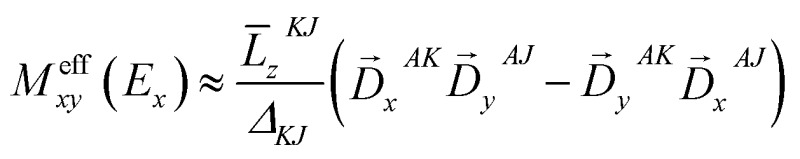


Thus, one can evaluate the MCD *C*-term sign in the saturation limit as follows,
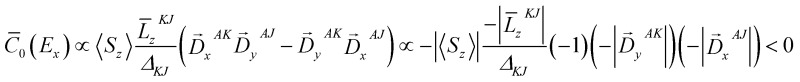
with*Δ*_*KJ*_ = *E*_*K*_ – *E*_*J*_ > 0.

Alternatively, the sign can be determined by a graphical approach.^12^ As shown in Fig. 4, the transition dipole moments and the reduced spin–orbit vector form a left-handed system, it follows that for positive *Δ*_*KJ*_ this will lead to absorption of right-handed photons, thereby resulting in a negative MCD *C*-term. In contrast, the MCD *C*-term signal of the higher-energy *E*_*y*_ transition has a positive sign.
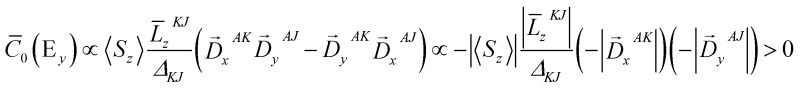
with *Δ*_*KJ*_ = *E*_*K*_ – *E*_*J*_ < 0. One may easily confirm this by swapping the EDO with the intermediate MO in Fig. 4. Taken together, the transitions *E*(d_*xz*,*yz*_ → d_*x* ^2^–*y* ^2^_) give rise to a positive pseudo-*A* term signal (the sign of a pseudo *A*-term is defined as that of the higher energy *C*-term signal) and are therefore assigned to bands 2 and 3. For completeness, the analysis of the MCD *C*-term sign of band 1 is documented in the ESI.†

Based on the computed excitation energies and the determined polarization, we tentatively assign band 4 to the ^3^ *E*(2e → 2a_1_) transitions and not to ^3^ *E*(1b_2_ → 2e), the latter of which is predicted to have quite low intensity because the EDO 1b_2_ is a nonbonding pure 3d-orbital. Using the same approach, we can predict that the pseudo-*A* term arising from the ^3^ *E*(2e → 2a_1_) transitions has a negative sign (Fig. S7†) as that delivered by the computations (Fig. 3). The *E*(d_*xz*,*yz*_ → d_*z* ^2^_) transitions have been observed in the MCD spectra of high spin [Fe^IV^(O)(TMG_3_tren)]^2+^ (TMG_3_tren = N[CH_2_CH_2_N=C(NMe_2_)_2_]) complex, and the sign of this pseudo-*A* term signal is also negative.^16*d*^ Due to the low intensity of this pseudo-*A* term signal, the adjacent intense CT transitions may substantially distort its band shape *via* out-of-state SOC and/or directly obscure it. Our theoretical results suggest that the onset of a series of the ligand-to-metal charge transfer (LMCT) transitions from the oxo group is around 22 430 cm^–1^; however, the dipole-allowed LMCT transition appears at ∼30 000 cm^–1^, which is significantly higher in energy than that of band 5. This is due to the lack of proper treatment of dynamic correlation, especially the radial correlation resulting from the change in the metal oxidation state in CT processes.^37^ To account for this effect, the active space has to be enlarged to include the metal double d-shell.^38^ Moreover, the intensity variation with different temperatures indicates band 5 to be largely *x*,*y*-polarized (Fig. S2†), while the dipole-allowed low-energy 1e → 2e LMCT transition must be *z*-polarized. Thus, LMCT transitions from the TPA ligand to the iron center may also contribute to this intense band. To compute the corresponding CT transitions, we need add the π-systems of TPA into the active space. Thus, the size of the active space is too large to make conventional CASSCF calculations impossible. Note that the present active space is large enough to deliver accurate excitation energies for ligand field transitions where static correlation prevails.^39^

### Complex **2**

#### ABS and MCD spectroscopy

**Fig. 5:**
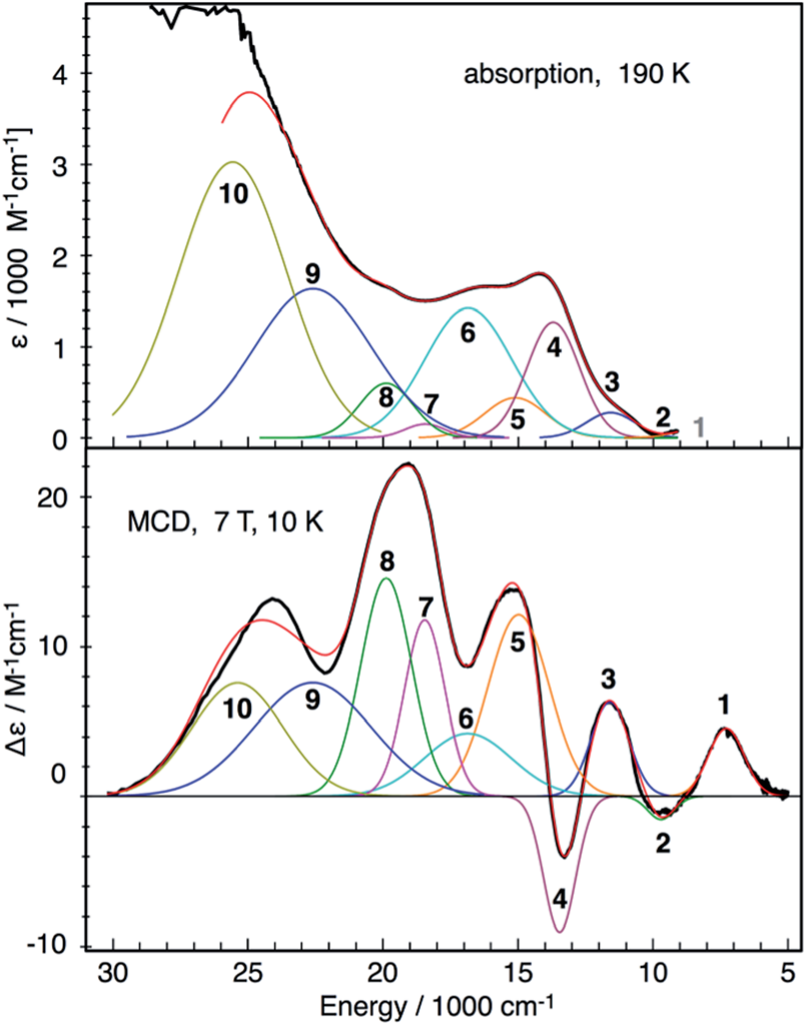
Electronic absorption and MCD spectra of complex **2** along with the global Gaussian deconvolution.

In contrast to complex **1**, complex **2** exhibits rich electronic transitions in the UV-vis region of the electronic absorption spectrum (Fig. 5, top). The presented spectrum was taken at 190 K; spectra recorded at even lower temperatures do not show significant differences but were of lower quality due to experimental limitations. Two major peaks are found to be centered at ∼14 000 cm^–1^ and ∼16 500 cm^–1^ with two shoulder bands at ∼11 000 cm^–1^ and ∼20 000 cm^–1^. In addition, one can identify the onset of one more transition below 10 000 cm^–1^, which cannot be fully established in the absorption spectrum due to the practical detection limit. This band (band 1) corresponds to the feature around 7200 cm^–1^ in the near infrared (NIR) MCD spectra (Fig. 5, bottom). The absorption and MCD spectra have been subjected to a global Gaussian deconvolution (Fig. 5) using the orca_asa program^40^ in which equal transition energies were enforced in the fitting (<3% shifts between absorption and MCD bands). At least ten Gaussian transitions are necessary in order to achieve a satisfactory fit of the MCD and absorption spectra. The fitting parameters are collected in Table 2. The calculated large *C*_0_/*D*_0_ values (>0.17) indicate bands 2–5, 7 and 8 to be d–d transitions in nature, whereas the remaining bands (6, 9, 10) are assigned to CT transitions.

**Table 2:** Spectral parameters obtained from the global Gaussian Fit of the absorption and MCD spectra of complex **2**

Band	Energy (cm^–1^)	% polarization (*x*, *y*, *z*)	*C*_0_/*D*_0_	Assignment
1	7280	32, 34, 34		d–d
2	9700	10, 82, 8	–0.95	d–d
3	11 600	1, 6, 93	0.21	d–d
4	13 460	14, 86, 0	–0.38	d–d
5	14 970	55, 45, 0	0.17	d–d
6	16 850	8, 0, 92	0.05	CT
7	18 450	9, 29, 71	0.24	d–d
8	19 880	29, 50, 21	0.19	d–d
9	22 600	32, 65, 3	0.04	CT
10	25 380	1, 9, 90	0.02	CT

#### Analysis of VTVH MCD data

**Fig. 6:**
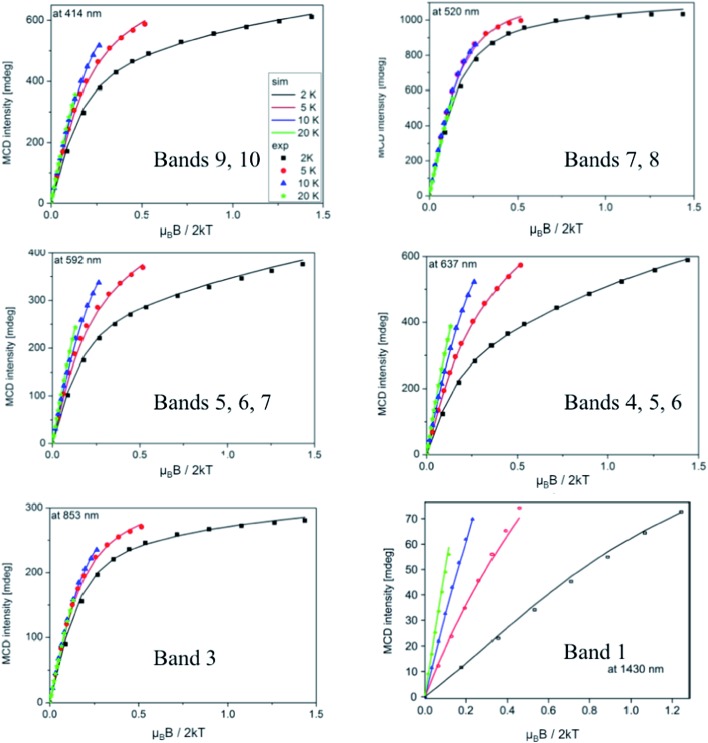
VTVH MCD magnetization curves recorded at 414, 520, 592, 637, 853 and 1430 nm (energies 24 160, 19 230, 16 890, 15 700, 11 720, 6990 cm^–1^) together with the best SH simulation.

MCD magnetization data of complex **2** are presented in Fig. 6, in which the dots represent experimental data collected at the selected wavelengths of 414, 520, 592, 637 and 853 nm as function of applied fields from 0 to 7 T at temperatures of 2, 5, 10 and 20 K. The data have been simulated by using a SH approach^12,41^ for a *ferromagnetically* exchange-coupled dimer of two local spins *S*_*i*_ = 1 with ZFS and exchange parameters as discussed below (Table S1†). As expected, the isothermal magnetization curves show significant nesting behavior, which indicates a considerable ZFS in the *S* = 2 ground state of complex **2**.

Chosen as the common reference frame for the fitting are the principal axes of the ZFS tensor for the Fe_A_ site, in which *Z* is parallel to Fe_A_–O_T_, *Y* is parallel to Fe_A_–O_B_, and *X* is parallel to the trans N–N direction.^42^ The local ZFS tensors in both sites was rotated by 90° according to the previous Mössbauer analyses^42^ and fixed in the fit. Systematic searches in the space of the SH parameters and the transition dipole moment products yielded a series of acceptable fits with nearly the same quality, as there are a number of local minima in this non-linear fitting procedure. Therefore, we constrained the value of *D*_A_ and (*E*/*D*)_A_ to those found previously for [Fe^IV^(O)(TPA)(NCMe)]^2+^ (*D* = +28 cm^–1^, *E*/*D* = 0).^8^ Even with these constraints, our simulations reveal that the error surface of the exchange constant *J* is rather flat; thus, it is not possible to obtain the *J* value with high precision. In fact, the choice of a *J* value in the range of 20–80 cm^–1^ does not discernibly deteriorate the quality of the fits. In this range of the *J* value, the fitted *D* parameter of the Fe_B_ site changes from 5.3 to 17.2 cm^–1^, while *E*/*D* is always close to 1/3. A satisfactory simulation is presented in Fig. 6 with following parameters: *g*_*x*,*y*,*z*_ = 2.00, *D*_B_ = +5.3 cm^–1^ (*E*/*D*)_B_ = 0.30 and *J* = 35 cm^–1^. The obtained SH parameters are in reasonable agreement with those determined independently by Mössbauer spectroscopy (Table 3), given the fact that the SH parameters are quite sensitive to the choice of *J*, a similar situation encountered by analyzing VTVH Mössbauer data.^42^

**Table 3:** Comparison of the SH parameters delivered by VTVH MCD and Mössbauer spectroscopy

	*D*_A_ (cm^–1^)	(*E*/*D*)_A_	*D*_B_ (cm^–1^)	(*E*/*D*)_B_	*J* (cm^–1^)
VTVH MCD	28	0	5.3–17.2	0.30	20–80
VTVH Mössbauer^42^	28	0	17.7	0.28	15–75^*a*^

^*a*^In our analysis, a different convention of the Heisenberg exchange Hamiltonian is employed *Ĥ*_ex_ = –2 ***Jŝ***_**A**_ **·*ŝ***_**B**_, in comparison with that used in the earlier Mössbauer study (*Ĥ*_ex_ = ***Jŝ***_**A**_ **·*ŝ***_**B**_). Thus, the values shown in Table 3 from the Mössbauer analysis have been converted from those reported in ref. 42.

To gain further information about the origin for each MCD band of complex **2**, the fractional polarization was determined by the VTVH MCD simulation based on the intensity of each band obtained by the Gaussian deconvolution (Fig. S8–11†).^43^ The obtained fractional polarizations in Table 2 suggest that bands 3, 4 and 6 are essentially *z*-, *y*- and *z*-polarized, respectively.

#### Electronic structure

**Fig. 7:**
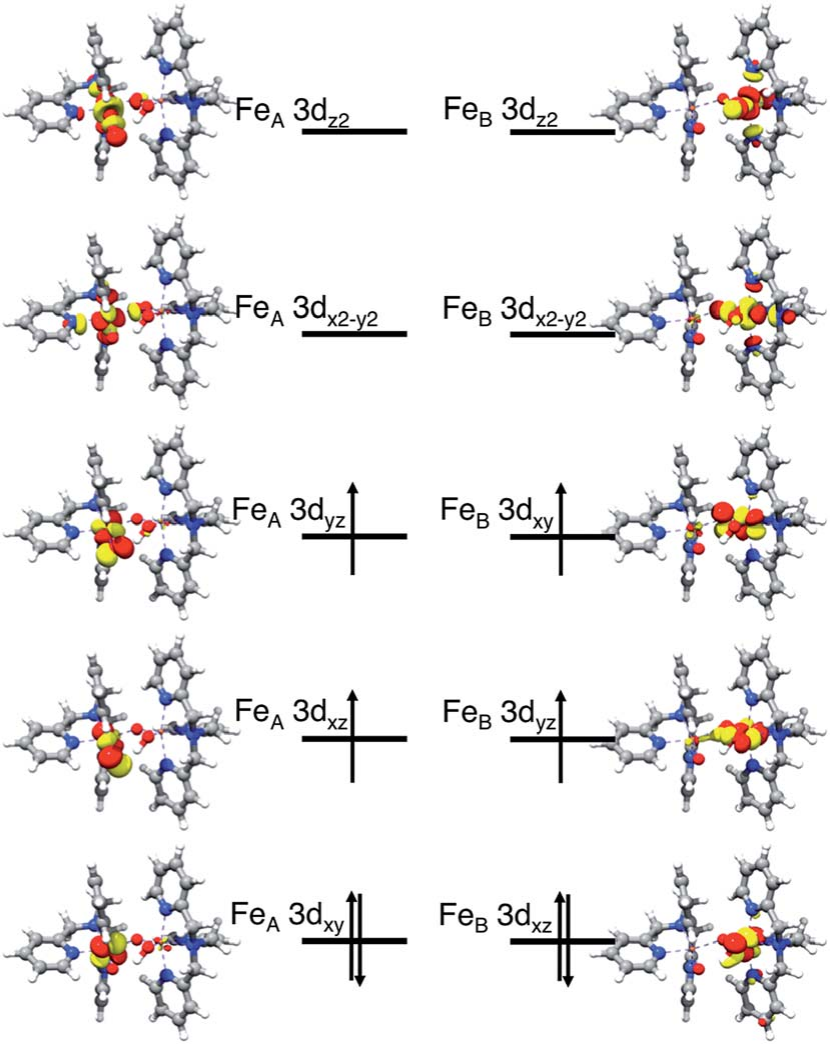
MO diagram of the ground state (configuration a) of complex **2** for which localized quasi-restricted orbitals obtained from the B3LYP calculations are employed.

Complex **2** contains a (μ-oxo)diiron(iv) core where one of the two iron(iv) centers is bound to a terminal oxo ligand (Fe_A_ =O) and the other is coordinated to a terminal hydroxo group (Fe_B_–OH); Fe_B_–OH forms a hydrogen bond with Fe_A_ =O (Scheme 1). In the optimized geometry the Fe_A_–O_T_ distance is calculated to be 1.639 Å, the Fe_A_–Fe_B_ distance 3.320 Å and the Fe_A_–O_B_–Fe_B_ angle 130.9°. These metric parameters match the EXAFS data^8^ (1.66 Å, 3.32 Å and 131°) very well. As analyzed previously,^42^ complex **2** features a quintet ground state arising from ferromagnetic coupling between the two *S* = 1 iron(iv) sites. To be consistent, we chose the same reference frame, the principal axes of the Fe_A_ ZFS tensor, to label the d-orbitals of the two iron sites. Since the angle of Fe_A_–O_B_–Fe_B_ is ∼130°, *Z* is roughly parallel to Fe_B_–O_H_. The geometric structure of the Fe_A_ site closely resembles that of complex **1** except that the equatorial MeCN is replaced by the bridging oxo group. In fact, the computed Fe_A_–O_B_ distance (1.931 Å) is close to the Fe–N_MeCN_ distance (1.982 Å) calculated for complex **1**, indicating much weaker bonding between Fe_A_ and O_B_ than one expected. For comparison, Collins and co-workers reported Fe–O_b_ distances of 1.73–1.74 Å for the only crystallographically characterized (μ-oxo)diiron(iv) complex to date.^44^ In line with this, the Fe_A_-d_*xy*_ based MO is a weakly π-antibonding orbital and is hence doubly occupied. Therefore, the electron configuration of Fe_A_ site is δ(Fe_A_-d_*xy*_)^2^π*(Fe_A_-d_*xz*,*yz*_)^2^σ*(Fe_A_-d_*x* ^2^–*y* ^2^_)^0^σ*(Fe_A_-d_*z* ^2^_)^0^ (Fig. 7), which is identical to that found for complex **1**. By contrast, the Fe_B_–O_B_ interaction is much stronger than Fe_A_–O_B_ as evidenced by the considerably shorter Fe_B_–O_B_ distance (1.730 Å). This bond length is also shorter than that of Fe_B_–O_OH_ (1.814 Å), reflecting the difference in Lewis basicity between oxo and hydroxo groups. Thus, the Fe_B_-d_*xy*_-based MO lies highest in energy among the three Fe_B_ *t*_2g_-derived MOs and is thus singly occupied. Our calculations show that there exist two alternative electron configurations for complex **2** depending on the relative energies of the two remaining Fe_B_ *t*_2g_-derived MOs:^42^ (a) π*(Fe_B_-d_*xz*_)^2^π*(Fe_B_-d_*yz*_)^1^π*(Fe_B_-d_*xy*_)^1^σ*(Fe_B_-d_*x* ^2^–*y* ^2^_)^0^σ*(Fe_B_-d_*z* ^2^_)^0^ (shown in Fig. 7) and b) π*(Fe_B_-d_*yz*_)^2^π*(Fe_B_-d_*xz*_)^1^π*(Fe_B_-d_*xy*_)^1^σ*(Fe_B_-d_*x* ^2^–*y* ^2^_)^0^σ*(Fe_B_-d_*z* ^2^_)^0^ with the former configuration lying ∼2 kcal mol^–1^ lower in energy. For configuration a), because the Fe_B_–O_OH_–H angle (106°) is close to a right angle, the O_OH_-p_*x*_ orbital cannot interact strongly with the Fe_B_-d_*xz*_ orbital but instead predominantly interacts with the H-s fragment orbital. As a result, the Fe_B_-d_*xz*_ based MO becomes stabilized to some extent relative to the Fe_B_-d_*yz*_ centered MO. In configuration (b) the Fe_B_–O_OH_–H angle is ∼140°, this lowers the energy of the Fe_B_-d_*yz*_ based MO.

#### Assignment

The calculated MCD spectrum of complex **2** (Fig. S12†) is overall in reasonable agreement with the experiment, which successfully reproduces the intense features (band 4–8) observed in the experimental MCD spectrum. According to the calculated transition energies for complex **2** (Table 1), we can safely assign band 1 as the lowest-energy ligand field *t*_2g_ → d_*x* ^2^–*y* ^2^_ transition of Fe_B_, and bands 2 and 3 as either Fe_B_ *t*_2g_ → d_*x* ^2^–*y* ^2^_ or Fe_A_ d_*xy*_ → d_*x* ^2^–*y* ^2^_, although our calculations underestimate the intensity of these weak d–d transitions. The positive MCD sign and the *z*-polarization of band 3, both of which are observed for the d_*xy*_ → d_*x* ^2^–*y* ^2^_ transition of complex **1**, further support the latter assignment. The lower excitation energy of band 3 than the corresponding transition found for complex **1** is congruent with the fact that for complex **2** the EDO Fe_A_-d_*xy*_ is a weakly π-antibonding orbital, while it is a nonbonding orbital for complex **1**. Based on the calculated excitation energies, the intense pseudo-*A* term features 4 and 5 can be assigned as the Fe_A_ d_*xz*,*yz*_ → d_*x* ^2^–*y* ^2^_ transitions. In contrast to complex **1**, the Fe_A_ d_*yz*_-based EDO of complex **2** is destabilized compared to Fe_A_ d_*xz*_ due to the additional π-antibonding interaction with O_B_; hence, the transition Fe_A_ d_yz_ → d_*x* ^2^–*y* ^2^_ lies at a lower energy. This interpretation is consistent with the determined polarization of band 4. It is important to note that, in principle, the sign of a pseudo-*A* term is independent of the relative order of the two constituent transitions.^12^ Thus, the pseudo-*A* term sign of d_*xz*,*yz*_ → d_*x* ^2^–*y* ^2^_ for complex **2** is predicted to be the same as that for complex **1**, which is indeed the case. The calculated MCD spectrum further corroborates this prediction (Fig. S12†). Bands 3, 4 and 5 can simultaneously acquire intensity at 2–10 K, as complex **2** possesses a substantially smaller *D* value (∼4 cm^–1^) along with significant rhombicity (*E*/*D* > 0.3).^42^ The smaller *C*_0_/*D*_0_ ratio of band 6 leads us to assign this band as a CT transition. Our calculations suggest that it arises either from the bridging oxo group to the Fe_B_ site or from the terminal OH group to Fe_B_. The latter assignment agrees with the polarization of band 6 deduced from the VTVH analysis. Based on the computed excitation energies, we tentatively assign bands 7 and 8 as the *t*_2g_ → d_*z* ^2^_ transitions of Fe_A_ and Fe_B_ sites. Given the *C*_0_/*D*_0_ ratio values, bands 9 and 10 are certainly CT transitions from the terminal oxo and hydroxo groups and/or the π-systems of TPA ligand to both iron centers. Our computations predict the wrong signs of bands 9 and 10. This may be attributed to the unbalanced treatment of the dynamic and static correlation in CT processes, similar to the situation encountered for band 5 of complex **1**.

### Correlation of the electronic structures with the reactivities

**Fig. 8:**
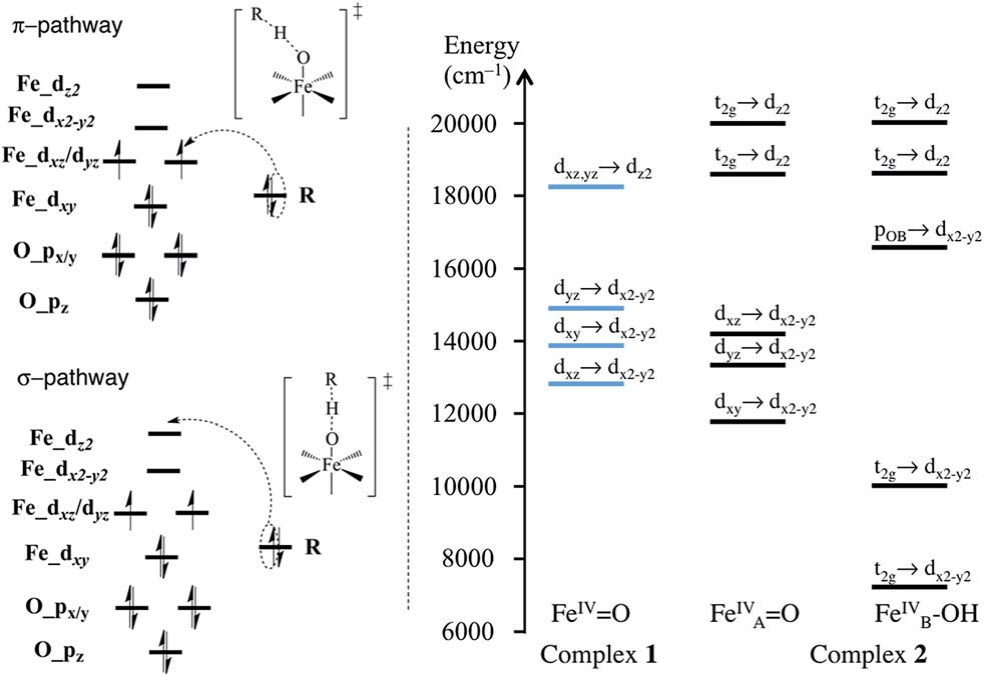
The electron transfer diagrams of the triplet π- and σ-pathways for H-atom transfer (left) and comparison of the excited state energies of complexes **1** and **2** (right).

H-atom transfer (HAT) is found to be the rate-determining step for C–H bond oxidation by iron(iv)-oxo complexes, which involves the transfer of an electron from the σ_C–H_ bond that is cleaved to the d_*xz*/*yz*_- (π-pathway) or d_*z* ^2^_-based MOs (σ-pathway) of the ferryl center and proton transfer to the oxo group (Fig. 8).^35b,45^ In comparison with complex **1**, the relevant d_*xz*,*yz*_ → d_*x* ^2^–*y* ^2^_ and d_*xz*,*yz*_ → d_*z* ^2^_ transitions of the Fe_A_ site in complex **2** have nearly identical excitation energies, differing by less than 1000 cm^–1^ (Fig. 8). This indicates that the interaction with another iron(iv) site does not discernibly change the local electronic structure of the reactive iron(iv)-oxo unit in complex **2**, consistent with the weak exchange interaction between the two Fe(iv) centers in complex **2**. Moreover, the local coordination environments of the Fe(iv)=O moiety in both complexes are similar, as they are supported by the same auxiliary ligand and the interaction between Fe_A_ and the bridging oxo ligand is relative weak (*vide supra*). Thus, one can anticipate that both complexes may suffer from similar energetic penalties arising from the electronic structure rearrangement and the resulting geometric distortions in the HAT process. As such, subtle effects such as hydrogen bonding may have a significant influence on the relative reactivities of the two complexes. Our previous work showed that the one-electron reduced form of complex **2** ([(L)Fe^IV^(O)(μ-O)Fe^III^(OH)(L)]^3+^) is 10-fold less reactive toward H-atom transfer than [(L)Fe^IV^(O)(μ-O)Fe^III^(F)(L)]^3+^, both of which share a similar core structure and feature the same electronic structure.^46^ The differential reactivity was attributed to the presence of a hydrogen bond between the terminal hydroxo and oxo groups in the former complex similar to that found for complex **2**, which entails an additional activation barrier for breaking this hydrogen bond during the reaction. Correspondingly, the experimental finding that the HAT reaction rate by complex **1** is five times faster than that by complex **2** can be readily rationalized.^9*c*^

Interestingly, the O-atom transfer (OAT) reactivities do not follow the trend of HAT, but were experimentally found to be the same between the two complexes.^9*c*^ Several experimental^47^ and theoretical investigations^48^ have revealed that OAT reactivity correlates strongly with the electron affinity of the iron(iv)-oxo species, whereas in the case of HAT, both the electron affinity of the oxidant and the proton affinity of its one-electron reduced form govern the reaction rate.^47,48^ Complex **2** has an overall +3 charge *versus* a +2 charge for **1**. The greater electron affinity of **2** due to its increased charge may then compensate for the added barrier required to break the hydrogen bond, to afford an OAT rate comparable to that of **1**. On the other hand, for an HAT reaction, the increased electron affinity of an oxidant also leads to a decreased proton affinity,^48*a*^ so the two factors cancel each other out and the effect of the hydrogen bond is observed.

## Summary

A detailed investigation of the electronic structures of a mono- (**1**) and a di-nuclear (**2**) iron(iv) complex supported by the same tetradentate tripodal ligand using a combined experimental and theoretical approach is presented herein. The salient features of the current work are the direct calculation of MCD spectra with wave-function-based multi-reference methods and independent determination of the MCD signs based on the associated EDO and EAO for a given transition. In comparison with experiment, this approach allows us to make unambiguous assignments of the important transitions of complex **1** and gain more insight into the MCD signs and the temperature-dependent intensity variations, both of which aid in our interpretation of the more complicated MCD spectra of complex **2**. Based on MCD/ABS intensity ratios, calculated excitation energies, polarizations, and MCD signs, the key transitions of complex **2** are assigned as ligand-field- or oxo- or hydroxo-to-metal charge transfer transitions. The correlation of the electronic structures of complexes **1** and **2** with their reactivities toward C–H bond oxidation and O-atom transfer reveals that, despite a difference in nuclearity, the two ferryl sites actually have very similar electronic structures that lead to similar reactivities.
