# Supplementary material for: Magnetic circular dichroism and computational study of mononuclear and dinuclear iron(iv) complexes
Source: Chem Sci. 2015 Feb 26;6(5):2909–21. doi: 10.1039/c4sc03268c (PMC4583211; doi:10.1039/c4sc03268c)
Supplement: Supplementary file 1 [file SC-006-C4SC03268C-s001.pdf]

Electronic Supplementary Material (ESI) for Chemical Science.
This journal is © The Royal Society of Chemistry 2015

SUPPORTING INFORMATION

Magnetic circular dichroism and computational study of mononuclear and dinuclear iron(IV) complexes

Shengfa Ye,[‡] Genqiang Xue,[&] Itana Krivokapic,[‡] Taras Petrenko,[‡] Eckhard Bill,[‡] Lawrence Que Jr.,[&] Frank Neese[‡]

[‡]Max-Planck Institut für Chemische Energiekonversion, Stiftstraße 34-36, D-45470 Mülheim an der Ruhr, Germany
[&]Department of Chemistry and Center for Metals in Biocatalysis, University of Minnesota, 207 Pleasant St. SE, Minneapolis, Minnesota 55455, United States

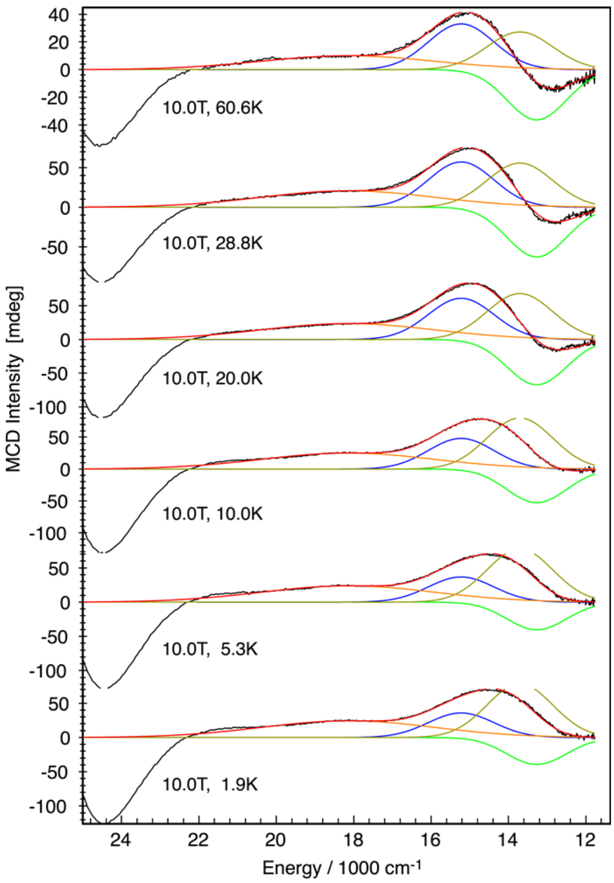

Figure S1. MCD spectra of complex **1** recorded at 10 T and indicated temperatures.

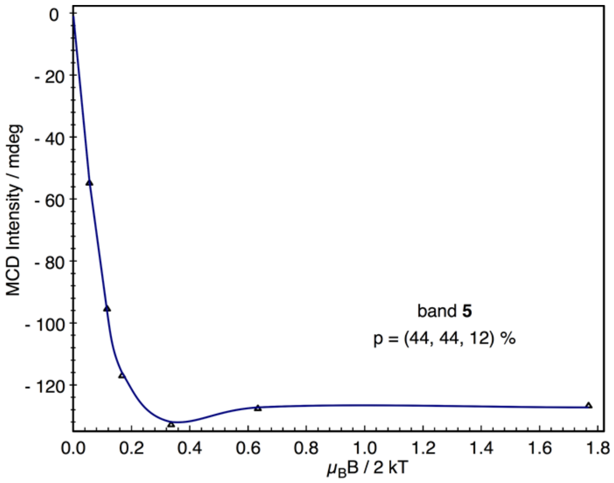

Figure S2. Temperature dependence of the intensity of band 5 at 24200 cm^{-1} of complex **1** as obtained from MCD spectra recorded at 10 T (Figures S1). The lines are derived from a spin-Hamiltonian simulation with $D = 28$ cm^{-1}, $E/D = 0$, and fractional polarization factors as indicated.

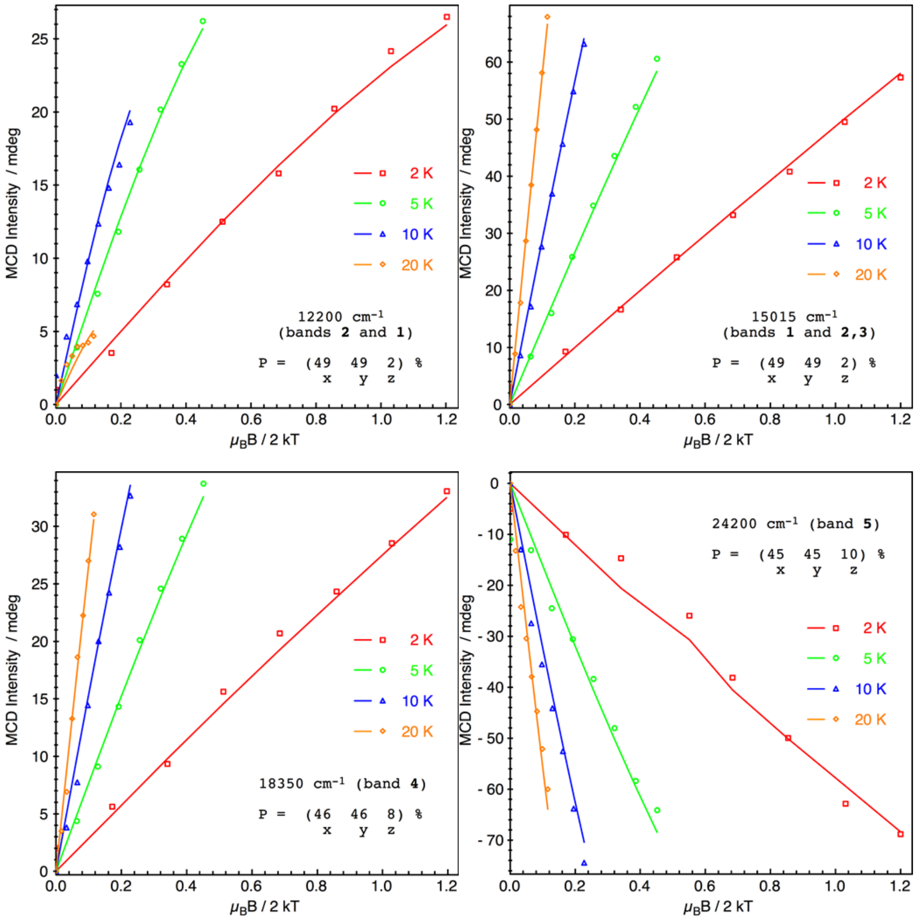

Figure S3. VTVH MCD magnetization data (dots) recorded at distinct temperatures (2, 5, 10, and 20 K) and distinct energies (12200, 15015, 18350, and 24200 cm^{-1}) together with global SH simulations (lines) with parameters D = 28 cm^{-1}, E/D = 0. The effective transition dipole moment products M_{vw}^{eff} obtained from the global parameter optimization yield fractional polarization factors as indicated in the insets.

Energies, $\langle S_x \rangle$,$\langle S_z \rangle$ values and Boltzmann populations of S = 1 magnetic sublevels as a function of the applied magnetic field.

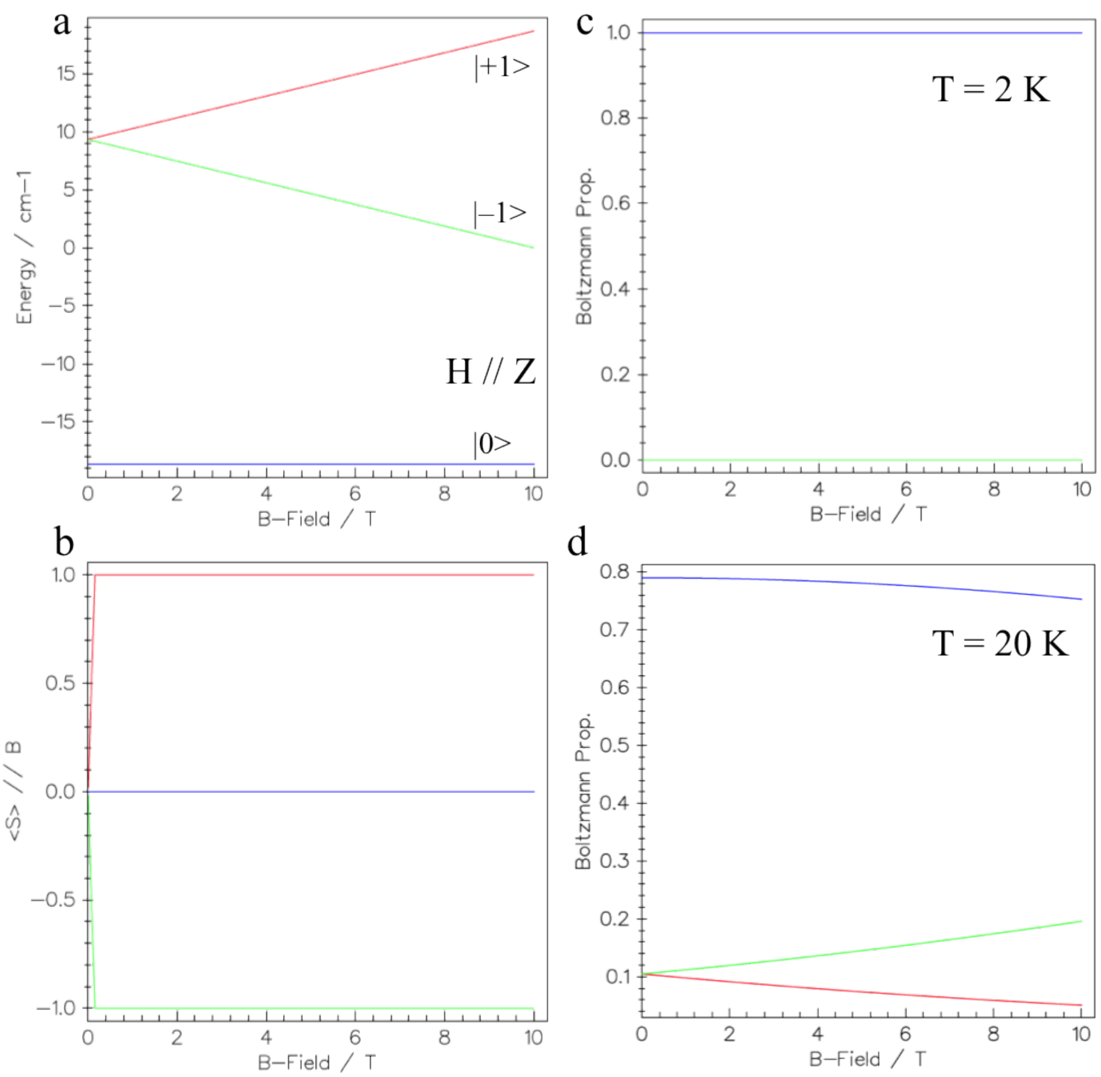

Figure S4. The energies (a), the expectation value of $\langle S_z \rangle$ (b) and the corresponding Boltzmann populations (c and d) of S = 1 magnetic sublevels as a function of the applied magnetic field for a system with $g_{x,y,z}$ = 2.0, D = 28 cm^{-1} and E/D = 0.

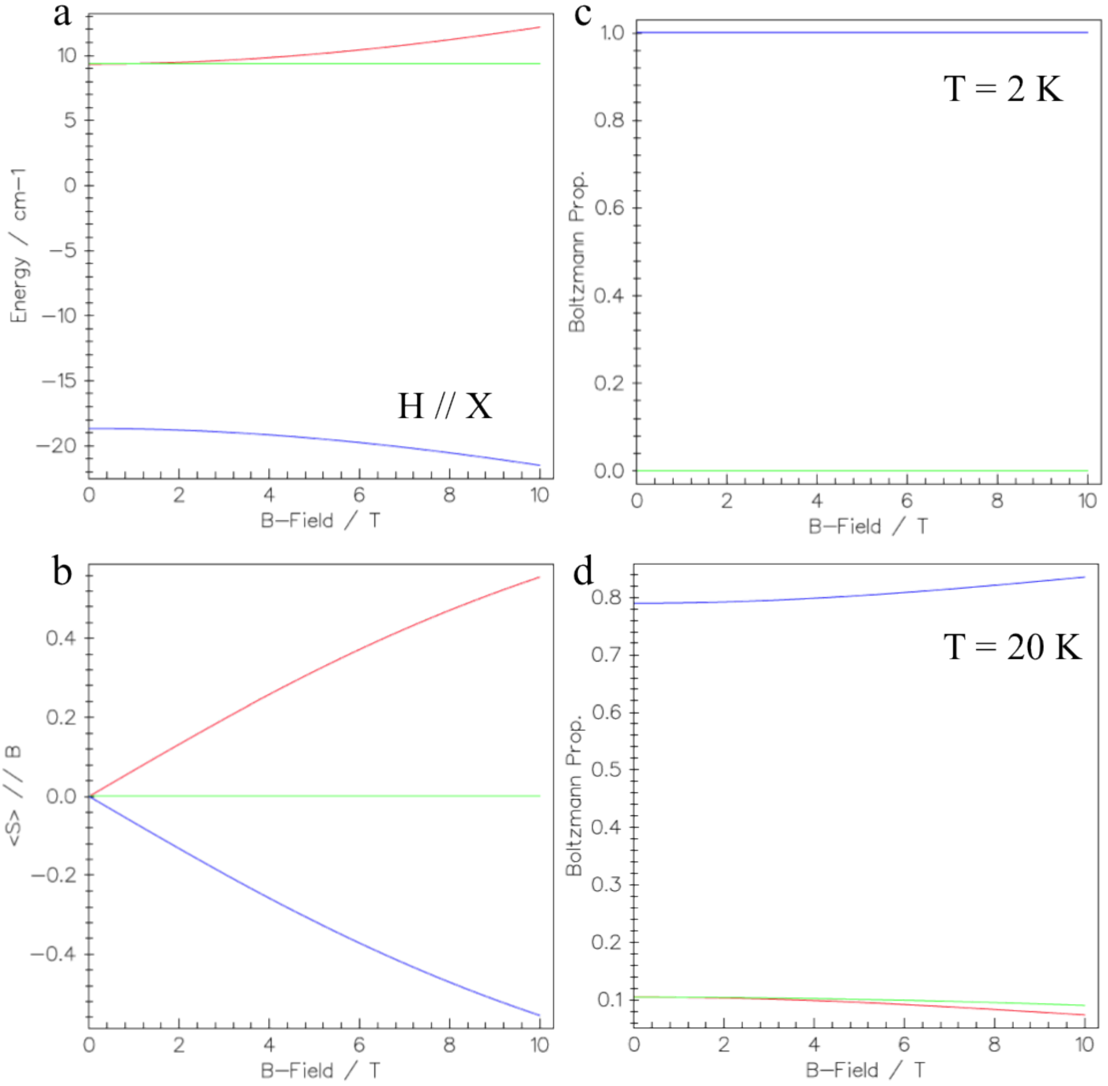

Figure S5. The energies (a), the expectation values of $\langle S_x \rangle$ (b) and the corresponding Boltzmann populations (c and d) of S = 1 magnetic sublevels as a function of the applied magnetic field for a system with $g_{x,y,z}$ = 2.0, D = 28 cm^{-1} and E/D = 0.

Derivation of the excited states arising from the $1b_2 \rightarrow 2b_1$ transition of complex **1**

In our CASSCF/NEVPT2 calculations, we found five low-lying excited states arising form the $1b_2 \rightarrow 2b_1$ transition, for which the corresponding electron configurations are shown in Figure S6. In configurations A and B, one of the 2e orbitals is doubly occupied, the linear combinations of these two configurations yield the excited states of symmetry A_2 and B_2, respectively.

$$|^3A_2\rangle = \frac{1}{\sqrt{2}}\left(|e_x\bar{e}_x b_2 b_1| + |e_y\bar{e}_y b_2 b_1|\right)$$

$$|^3B_2\rangle = \frac{1}{\sqrt{2}}\left(-|e_x\bar{e}_x b_2 b_1| + |e_y\bar{e}_y b_2 b_1|\right)$$

The linear combinations of configurations C, D, E and F generate three triplet excited states of $^3B_1$ and $^3A_1(\times 2)$ symmetry, respectively, in addition to the $M_s = 1$ component of the spin-flip excited state $^5A_1$.

$$|^3B_1\rangle = \frac{1}{\sqrt{2}}\left(|e_x\bar{e}_y b_2 b_1| + |e_y\bar{e}_x b_2 b_1|\right)$$

$$|^3A_1\rangle = \frac{1}{\sqrt{6}}\left(2|e_x e_y \bar{b}_2 b_1| - |\bar{e}_x e_y b_2 b_1| - |e_x\bar{e}_y b_2 b_1|\right)$$

$$|^3A_1\rangle = \frac{1}{\sqrt{12}}\left(3|e_x e_y b_2 \bar{b}_1| - |\bar{e}_x e_y b_2 b_1| - |e_x\bar{e}_y b_2 b_1| - |e_x e_y \bar{b}_2 b_1|\right)$$

$$|^5A_1, M_s = 1\rangle = \frac{1}{2}\left(|\bar{e}_x e_y b_2 b_1| + |e_x\bar{e}_y b_2 b_1| + |e_x e_y \bar{b}_2 b_1| + |e_x e_y b_2 \bar{b}_1|\right)$$

On the basis of the above wave-functions, we can compute the energy differences among them as follows,

$$E(^3A_2) - E(^3B_2) = 2K(ee)$$

$$E(^3A_2) - E(^3B_1) = J'(ee) - J(ee)$$

$$E(^3A_2) - \bar{E}(^3A_1) = J'(ee) - J(ee) + 2K(ee) - \frac{1}{2}K(eb_1) - \frac{1}{2}K(eb_2) - K(b_1b_2)$$

$\bar{E}(^3A_1)$ is the average energy of the two $^3A_1$ state, $J'(ee) = \left\langle e_x e_x \middle| \frac{1}{r_{1,2}} \middle| e_x e_x \right\rangle = \left\langle e_y e_y \middle| \frac{1}{r_{1,2}} \middle| e_y e_y \right\rangle$ is the on-site Coulomb integral and $J(ee) = \left\langle e_x e_y \middle| \frac{1}{r_{1,2}} \middle| e_x e_y \right\rangle$ is the inter-site Coulomb integral.

The 2e orbitals contain considerable O-p character and the covalency leads to stronger reduction in the on-site Coulomb integral than the inter-site one. Thus, one cannot determine *a priori* the relative energies among these excited states without carrying out calculations. Our calculations in fact show that all these excited state have similar

transition energies (13000, 13170, 14150, 16440, 18370 cm^{-1}) differing by ~ 5000 cm^{-1}.

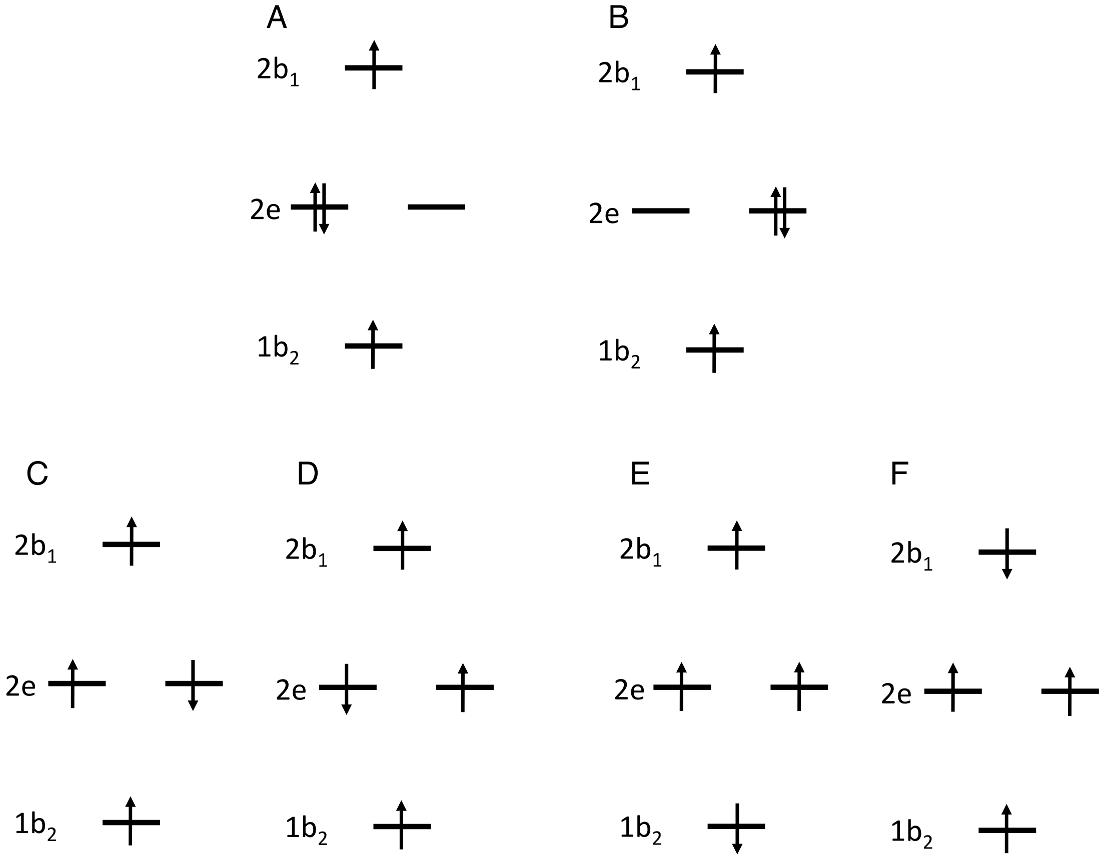

Figure S6. Low-lying electron configurations involves in the $1b_2 \rightarrow 2b_1$ transition.

For the transition $^3E(2e \rightarrow 2b_1)$

$|^3E_x\rangle = |b_2\bar{b}_2 b_1 e_y|$, $|^3E_y\rangle = |b_2\bar{b}_2 e_x b_1|$

$$E(^3A_2) - E(^3E) = h(e) - h(b_2) + J'(ee) + K(ee) + J(eb_1) - J(b_1b_2) - J(b_2b_2)$$

For the transition $^3E(1b_2 \rightarrow 2e)$

$|^3E_x\rangle = |b_2\bar{e}_y e_x e_y|$, $|^3E_y\rangle = |b_2\bar{e}_x e_x e_y|$

$$E(^3A_2) - E(^3E) = h(b_1) - h(e) - 2J(ee) + 2K(ee) + 2J(eb_1) - K(eb_1)$$
$$-J(eb_2) + K(eb_2) + J(b_1b_2) - K(b_1b_2)$$

Determination of the MCD *C*-term sign of the $A_2(1b_2 \rightarrow 2b_1)$ transition

In the effective C_{4v} symmetry of complex **1**, the $1b_2 \rightarrow 2b_1$ transition results in the excited states of $A_1(\times 2)$, A_2, B_1 and B_2 symmetry, respectively. Only the transition to A_2 is dipole-allowed and polarized along the z-direction. However, because the actual symmetry of complex **1** is C_s, all five excited states are mixed and all transitions are symmetry-allowed. One excited state is computed to appear at 16440 cm^{-1}, which may contribute to the z-polarized transition around 17000 cm^{-1}.

Band 1 which is assigned as ${}^3A_2(1b_2 \rightarrow 2b_1)$ is in fact a two-electron transition and hence has vanishing intensity. Lowing the symmetry from C_{4v} to C_s allows band 1 to borrow intensity from intense transitions, which are close in energy. Thus, band 1 has no well-defined polarization property (x, y, z = 38%, 38%, 24%), as deduced from the VTVH analysis. The spin-orbit coupling (SOC) of ${}^3A_2(1b_2 \rightarrow 2b_1)$ with $E(2e \rightarrow 2b_1)$ provide a feasible source for the positive C-term signal of band 1 (see below). The SOC with other excited states, such as ${}^3E(1b_2 \rightarrow 2e)$, may also contribute to this positive *C*-term signal because of the complex polarization property of band 1. However, due to the low intensity, the absolute polarization of the transitions ${}^3E(1b_2 \rightarrow 2e)$ cannot be determined unambiguously, which hampers the further analysis.

Using the same approach to determine the MCD *C*-term sign of $E(2e \rightarrow 2b_1)$, we can show that the SOC between $A_2(1b_2 \rightarrow 2b_1)$ and $E(2e \rightarrow 2b_1)$ leads to the positive *C*-term MCD sign of band 1, if the transition energies of $E(2e \rightarrow 2b_1)$ are higher than that of $A_2(1b_2 \rightarrow 2b_1)$ as found experimentally.

If $J = {}^3A_2$, $K = {}^3E_x = |b_2\bar{b}_2 b_1 e_y|$

$$\bar{L}_y^{KJ} = \frac{1}{\sqrt{8}} Im\langle d_{yz} | \hat{l}_y | d_{xy}\rangle$$

Rotation of the d_{xy} orbital to d_{yz} along the y-direction results in a negative overlap, thus $\bar{L}_y^{KJ} < 0$.

$$\bar{C}_0(A_2) \propto \langle S_y\rangle \frac{\bar{L}_y^{KJ}}{\Delta_{KJ}}\left(\vec{D}_z^{AK}\vec{D}_x^{AJ} - \vec{D}_x^{AK}\vec{D}_z^{AJ}\right) \propto -|\langle S_y\rangle| \frac{-|\bar{L}_y^{KJ}|}{\Delta_{KJ}}(-1)\left(-|\vec{D}_x^{AK}|\right)\left(+|\vec{D}_z^{AJ}|\right) > 0$$

Similarly, if $J = {}^3A_2$, $K = {}^3E_y = |b_2\bar{b}_2 e_x b_1|$

$$\bar{L}_x^{KJ} = \frac{1}{\sqrt{8}} Im\langle d_{xy} | \hat{l}_x | d_{xz}\rangle > 0$$

$$\bar{C}_0(A_2) \propto \langle S_x\rangle \frac{\bar{L}_x^{KJ}}{\Delta_{KJ}}\left(\vec{D}_y^{AK}\vec{D}_z^{AJ} - \vec{D}_z^{AK}\vec{D}_y^{AJ}\right) \propto -|\langle S_x\rangle| \frac{+|\bar{L}_y^{KJ}|}{\Delta_{KJ}}\left(-|\vec{D}_y^{AK}|\right)\left(+|\vec{D}_z^{AJ}|\right) > 0$$

Determination of the MCD *C*-term sign of the $E(2e \rightarrow 2a_1)$ transitions

Similar to $E(2e \rightarrow 2b_1)$, one may expect another MCD pseudo-A term signal arising from the $E(d_{xz,yz} \rightarrow d_{z^2})$ transitions. Using the same approach, we can show that this pseudo-A term has a negative sign (Figure S7), just opposite to that of $E(d_{xz,yz} \rightarrow d_{x^2-y^2})$. Unlike the $d_{x^2-y^2}$-centered EAO, the ligand N-p contributions in the d_{z^2}-based EAO have the same phase in the equatorial plane, so the transition dipole moment of $E_y(d_{yz} \rightarrow d_{z^2})$ points along the opposite direction (+y), but that of $E_x(d_{xz} \rightarrow d_{z^2})$ is unaffected (–x). This reverses the MCD *C*-term sign for the individual transition from the same EDO, and hence the sign of the entire pseudo-A term. Moreover, the intensity of the $E(d_{xz,yz} \rightarrow d_{z^2})$ transitions must be lower than that of $E(d_{xz,yz} \rightarrow d_{x^2-y^2})$, because the Fe-$d_{z^2}$ orbital predominantly interacts with the axial ligands and thus the equatorial ligand contributions in the d_{z^2} based MO have less weight compared to those in the $d_{x^2-y^2}$-centered MO. Our prediction about the signs and the intensities of the $E(d_{xz,yz} \rightarrow d_{z^2})$ transitions is consistent with the corresponding pseudo-A feature around 18000 cm^{-1} in the calculated MCD spectra.

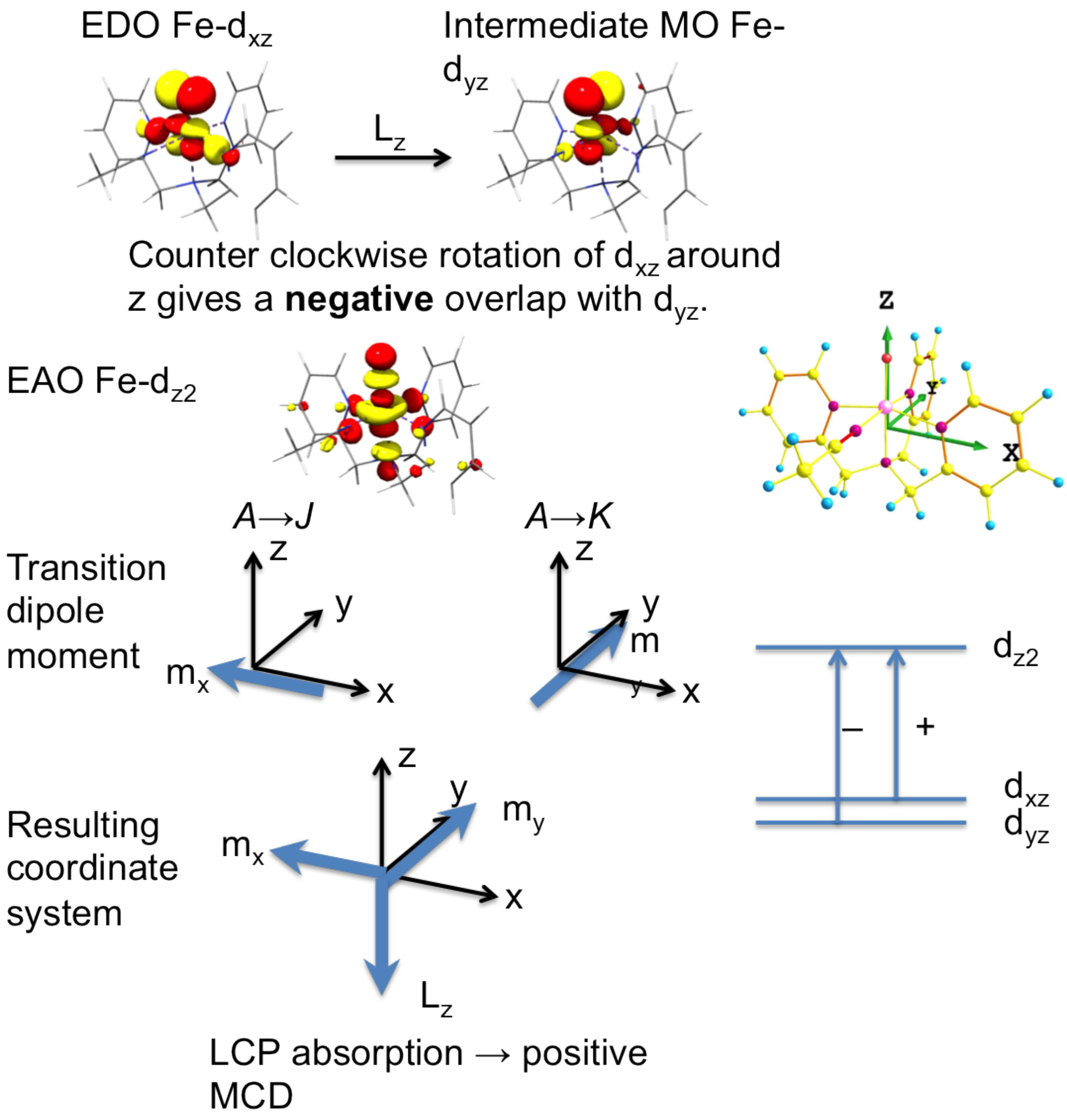

Figure S7. Graphical prediction of the *C*-term sign for the $d_{xz,yz} \rightarrow d_{z^2}$ transitions.

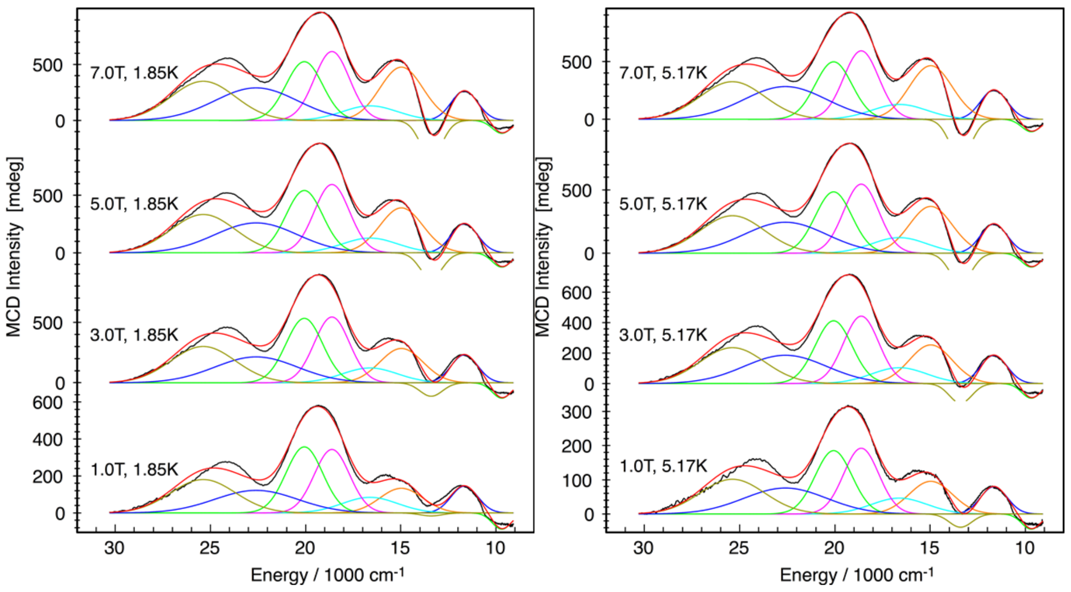

Figure S8. Field-dependent MCD spectra of complex **2** recorded at ca. 2 and 5 K.

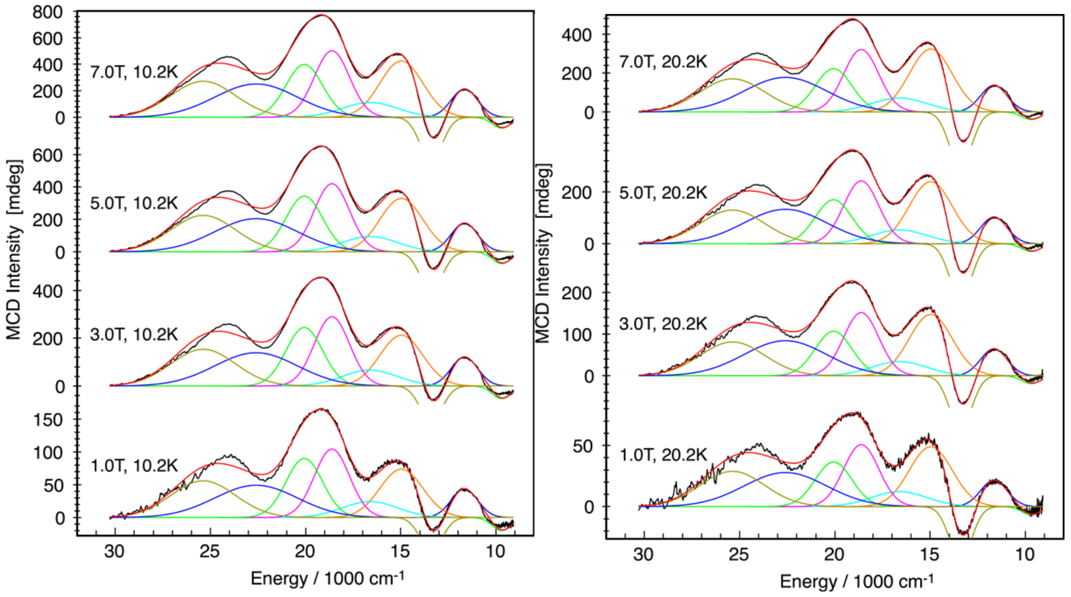

Figure S9. Field-dependent MCD spectra of complex **2** recorded at ca. 10 and 20 K.

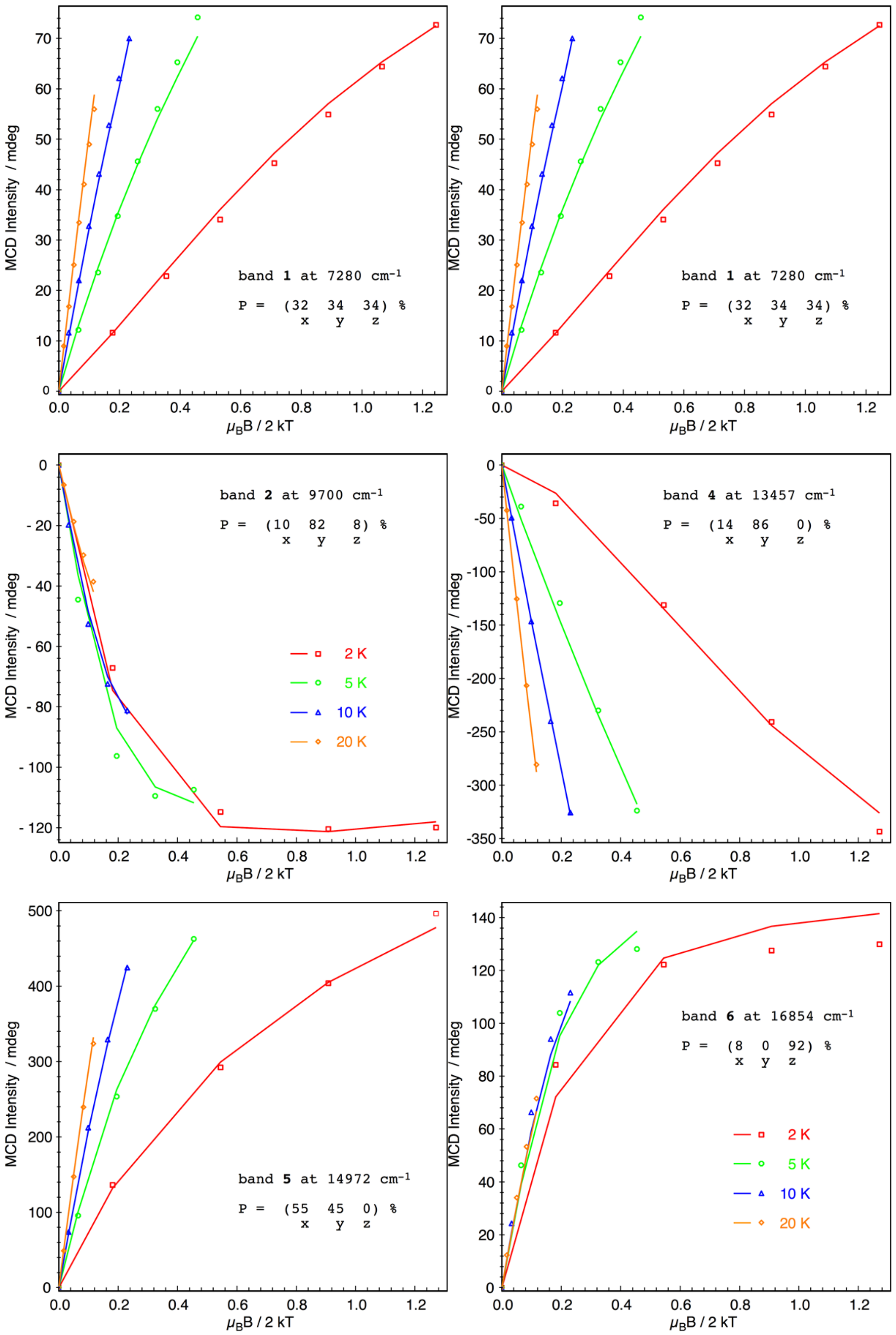

Figure S10. VTVH simulation of the intensities of the MCD band 1–6 of complex **2** obtained from the Gaussian deconvolution (Figures S8, S9), which yield the fractional polarization factors as given in Table 2. In the fit, the SH parameters were fixed with $g_{x,y,z} = 2.00$, $D_B = +5.3$ cm^{-1} $(E/D)_B = 0.30$ and $J = 35$ cm^{-1}.

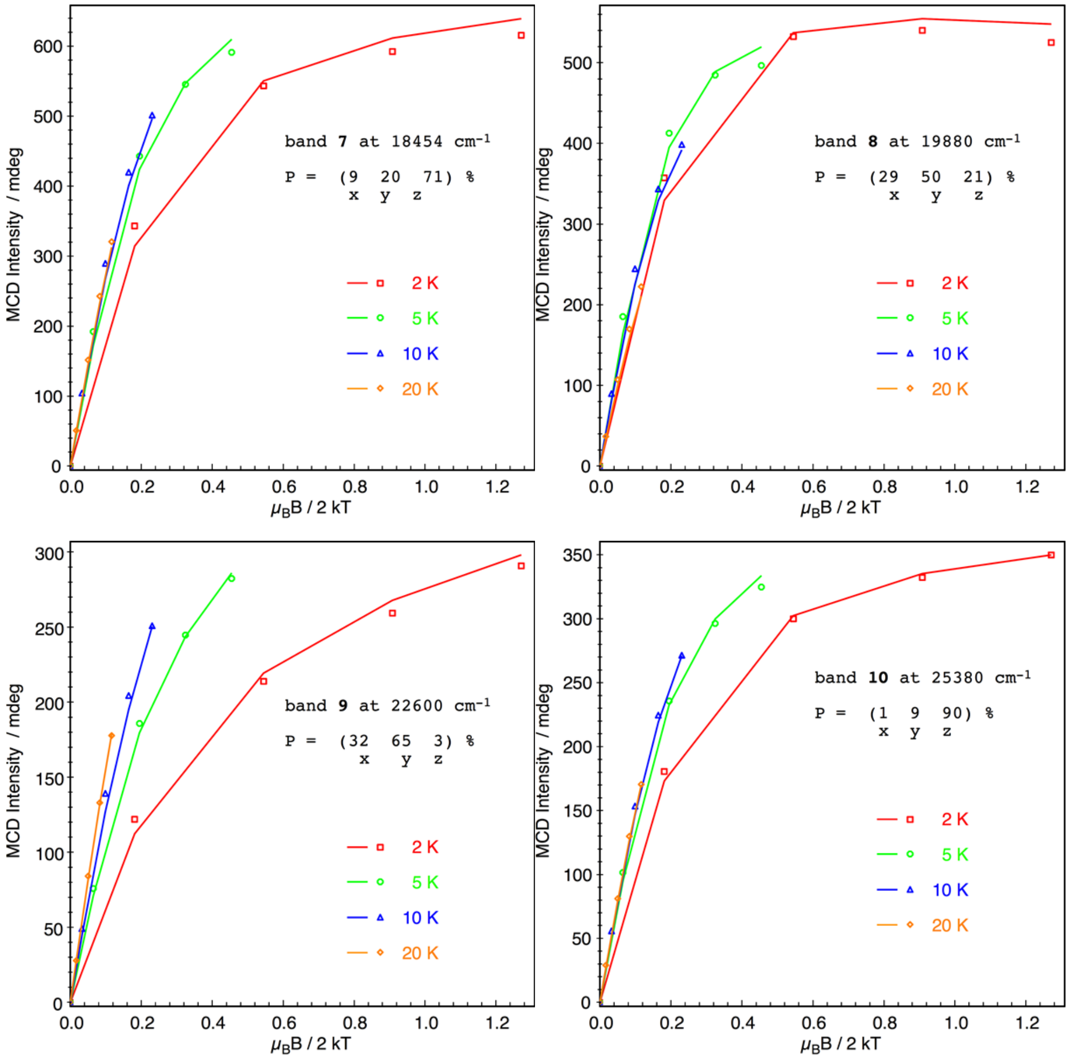

Figure S11. VTVH simulation of the intensities of the MCD band 7–10 of complex **2** obtained from the Gaussian deconvolution (Figures S8, S9), which yield the fractional polarization factors as given in Table 2. In the fit, the SH parameters were fixed with $g_{x,y,z} = 2.00$, $D_B = +5.3$ cm^{-1} $(E/D)_B = 0.30$ and $J = 35$ cm^{-1}.

Table S1. Computed Fractional Polarization Values of Key Transitions for Complex **2**.

	x	y	z
853 nm (11700 cm^{-1})	0.11	0.30	0.59
637 nm (15700 cm^{-1})	0.70	0.21	0.09
592 nm (16900 cm^{-1})	0.09	0.89	0.02
520 nm (19200 cm^{-1})	0.03	0.15	0.82
414 nm (24100 cm^{-1})	0.27	0.25	0.48

Calculated MCD spectrum of complex **2**

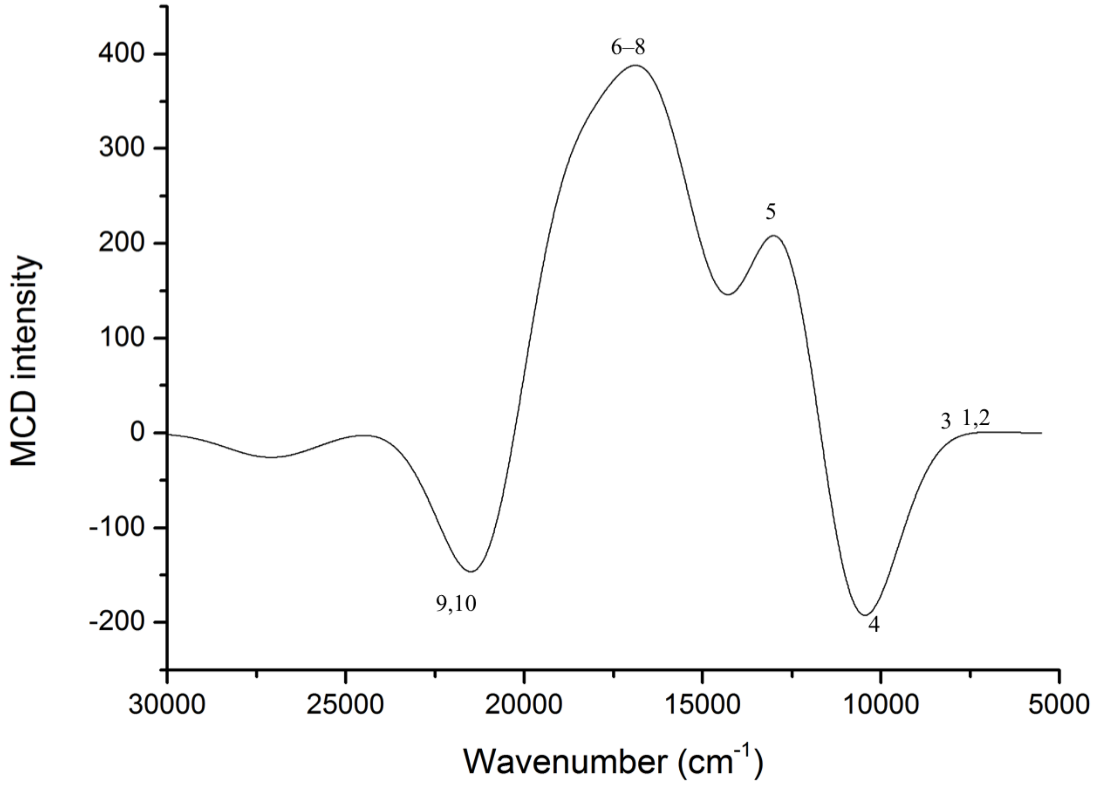

Figure S12. Computed MCD spectrum of complex **2**.
